# RanBP9 controls the oligomeric state of CTLH complex assemblies

**DOI:** 10.1016/j.jbc.2023.102869

**Published:** 2023-01-05

**Authors:** Pia Maria van gen Hassend, Aparna Pottikkadavath, Carolyn Delto, Monika Kuhn, Michelle Endres, Lars Schönemann, Hermann Schindelin

**Affiliations:** Julius-Maximilians-Universität Würzburg, Rudolf Virchow Center for Integrative and Translational Bioimaging, Institute of Structural Biology, Würzburg, Germany

**Keywords:** RING E3 ligase, SEC–MALS, ITC, X-ray crystallography, GID–CTLH complex, AR, armadillo repeats, aSEC, analytical size-exclusion chromatography, cat, Rmnd5a-Maea complex, CC, coiled-coiled region, CRA, CT-11-RanBPM, CRA^C^, C-terminal helix of the CRA domain, CRA^N^, N-terminal part of the CRA domain, CTLH, C-terminal to lissencephaly-1 homology motif, D, discoidin domain, EMDB, Electron Microscopy Data Bank, GID, glucose-induced degradation deficient, ITC, isothermal titration calorimetry, KR, kelch repeats, L/LisH, lissencephaly-1 homology, M, muskelin, Ma, Maea, SEC-MALS, multiangle light scattering coupled to aSEC, NAGE, native agarose gel electrophoresis, PDB, Protein Data Bank, R, RanBP9, R5, Rmnd5a, RING, really interesting new gene, RT_cat_, RanBP9–Twa1–Rmnd5a–Maea complex, SA, supramolecular assembly module, SPRY, SPla and the RYanodine receptor, SR, substrate receptor, SRS, SR scaffolding module, T, Twa1, W, Wdr26, WD, WD40 repeat, α, Armc8α, β, Armc8β

## Abstract

The CTLH (C-terminal to lissencephaly-1 homology motif) complex is a multisubunit RING E3 ligase with poorly defined substrate specificity and flexible subunit composition. Two key subunits, muskelin and Wdr26, specify two alternative CTLH complexes that differ in quaternary structure, thereby allowing the E3 ligase to presumably target different substrates. With the aid of different biophysical and biochemical techniques, we characterized CTLH complex assembly pathways, focusing not only on Wdr26 and muskelin but also on RanBP9, Twa1, and Armc8β subunits, which are critical to establish the scaffold of this E3 ligase. We demonstrate that the ability of muskelin to tetramerize and the assembly of Wdr26 into dimers define mutually exclusive oligomerization modules that compete with nanomolar affinity for RanBP9 binding. The remaining scaffolding subunits, Armc8β and Twa1, strongly interact with each other and with RanBP9, again with nanomolar affinity. Our data demonstrate that RanBP9 organizes subunit assembly and prevents higher order oligomerization of dimeric Wdr26 and the Armc8β–Twa1 heterodimer through its tight binding. Combined, our studies define alternative assembly pathways of the CTLH complex and elucidate the role of RanBP9 in governing differential oligomeric assemblies, thereby advancing our mechanistic understanding of CTLH complex architectures.

Ubiquitylation of target proteins and their subsequent degradation is an important mechanism allowing the cell to react to different environmental stimuli and ensure proper cellular functions in eukaryotes. Ubiquitylation specificity is achieved by interactions of substrates with E3 ligases, which catalyze the last step in the ubiquitin transfer cascade (1). The RING (really interesting new gene) type E3 ligases constitute the largest group of ligases, which, in human, is comprised of more than 600 different members. A hallmark feature of this class is the presence of a RING domain, which binds ubiquitin-conjugating E2 enzymes, thereby bringing them into close proximity to the simultaneously bound substrate (2, 3, 4). The CTLH (C-terminal to lissencephaly-1 homology [LisH] motif) complex is a multisubunit RING E3 ligase, which is characterized by the recurrence of certain motifs or domains in its core subunits (5). These include the LisH (lissencephaly-1 homology) and CTLH motifs, which are present in the two RING subunits Rmnd5a and Maea, the scaffolding subunits RanBP9 and Twa1 and the oligomerization modules Wdr26 and muskelin as well as the CRA (CT-11-RanBPM) domain, which occurs in the RING domains and the scaffolding subunits (Fig. 1*A*). These recurring motifs endow this E3 ligase with the ability to homodimerize and heterodimerize and are critical to establish its overall architecture (6). The remaining subunits lack these domains but are important as substrate receptors (SRs) and their adaptors.

**Figure 1:**
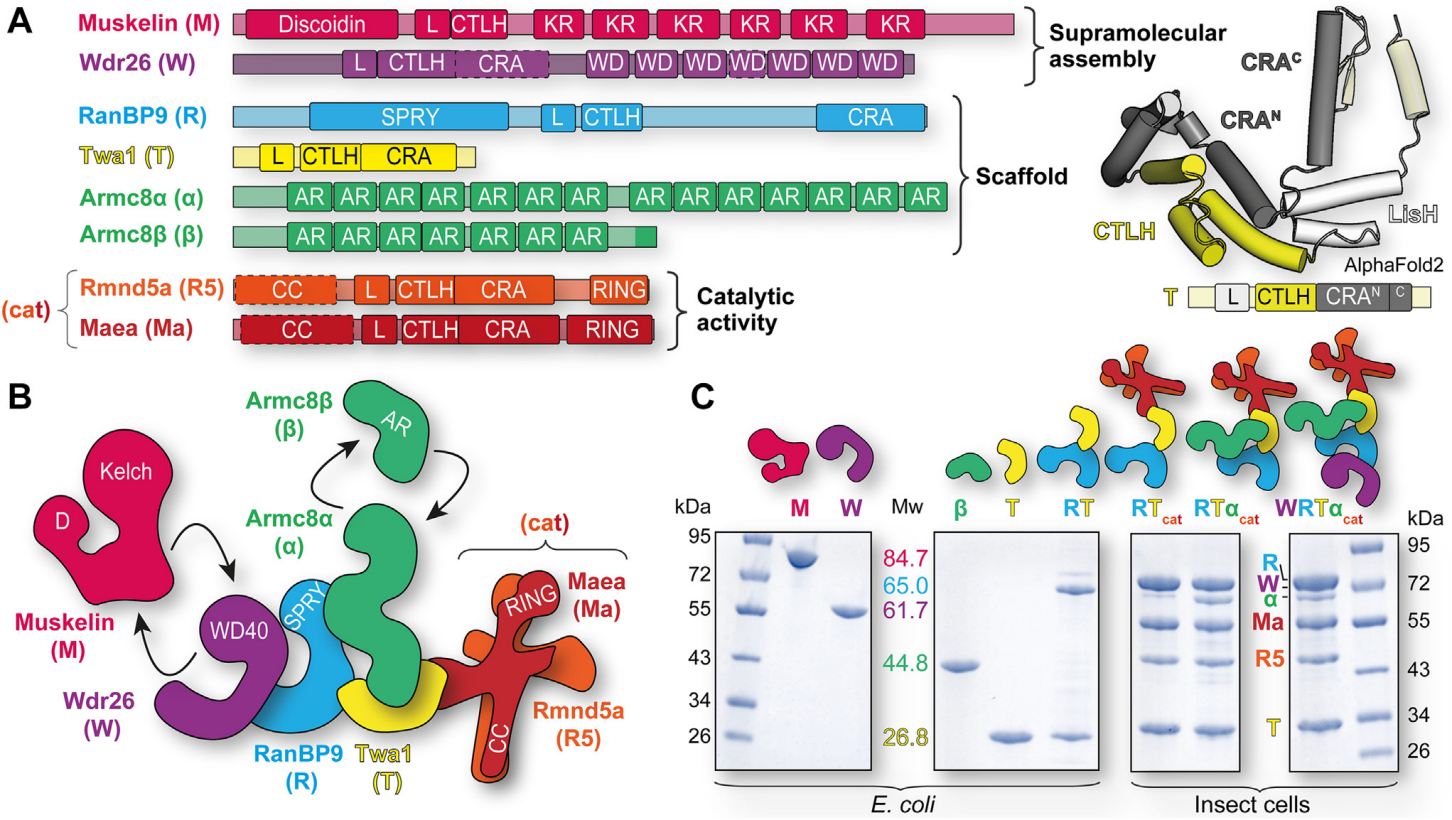
**Modular reconstitution of CTLH complexes.***A*, domain architecture of CTLH subunits based on the InterPro database and additional assignments based on AlphaFold2 predictions (dotted lines). Folding of the recurring LisH (L), CTLH, and CRA domain sequence is illustrated by the Twa1 AlphaFold2 model. *B*, model of the CTLH complex as defined earlier (6). Unique domains are highlighted, and arrows represent alternative subunits. *C*, SD-PAGE analyses of purified individual proteins and different CTLH subcomplexes after expression in either *Escherichia coli* or insect cells. Theoretical molecular weights (colored numerals with units in kilodalton) of the monomeric proteins as reference for the SEC–MALS analyses are indicated. AR, armadillo repeat; CC, coiled-coil region; CRA, CT-11–RanBPM; CTLH, C-terminal to lissencephaly-1 homology motif; D, discoidin; KR, kelch repeat; L, lissencephaly-1 homology; RING, really interesting new gene; SPRY, SPla and the RYanodine receptor; SEC-MALS, multiangle light scattering coupled to analytical size-exclusion chromatography; WD, WD40 repeat.

The CTLH complex is involved in numerous cellular pathways (7) including signaling processes like regulating sonic hedgehog components (8) at the primary cilium (9, 10) degradation of c-Raf in the extracellular signal–regulated kinase pathway (11), and CTLH subunits were also linked to the Wnt-signaling pathway (12, 13, 14). Dysregulation of CTLH components correlates with poor prognosis and tumor progression in various cancer types (15, 16, 17, 18, 19). CTLH activities have been further implicated in cell proliferation (20, 21), aging (22), metabolism (23, 24), and developmental processes. The complex is also responsible for an organized maternal-to-zygotic transition (25, 26) and is important for neurodevelopment (27, 28, 29, 30) as well as hematopoiesis (31, 32, 33, 34, 35, 36). While the name CTLH complex suggests that there is a single complex with a defined subunit architecture, there are in fact multiple alternative subunits, which may lead to differential CTLH complexes. A mechanistic understanding of how this variety of functions is achieved, such as the identification of substrates being targeted and their cognate SR subunit or domain within differential CTLH assemblies, remains elusive.

Insights into the principles governing substrate specificity of the CTLH complex were mostly derived from studies of the GID (glucose-induced degradation deficient) complex, the more extensively characterized yeast counterpart of the phylogenetically conserved (37) CTLH complex. In this system, substrates are recognized by selective expression of SRs upon different environmental stimuli. Targeting of superfluous gluconeogenic enzymes (*e.g.*, fructose-1,6-bisphosphatase, Fbp1) was the first identified activity for the GID complex as a catabolite inactivation mechanism after switching cells to glycolytic conditions (38, 39, 40, 41). Fbp1 is recognized *via* the SR subunit Gid4, which binds to N-terminal proline residues (N-degron) with its β-barrel architecture and serves as N-recognin in the proline (Pro)/N-degron pathway (1, 42, 43, 44, 45, 46). Gid4 is highly conserved from yeast to humans, presumably also targeting substrates following the Pro/N-degron pathway in the mammalian system; however, so far none have been identified. Notably, human Fbp1 is not targeted by the CTLH complex (20).

Alternative substrate recognition of the GID complex is achieved by different SRs such as Gid10, which can be substituted for the similarly folded Gid4 subunit. Gid10 acts in response to starvation, osmotic stress, and heat shock, with Rsp5 being the only substrate identified so far (23, 46, 47, 48, 49). A distinct third SR, Gid11, which is predicted to contain a WD40 repeat containing β-propeller domain, recognizes predominantly threonine residues instead of an N-terminal proline (1, 50). Substrate-targeting abilities of the GID complex are further modulated by the recently identified Gid12 subunit that restricts substrate binding by obstructing the Gid4-binding site (51). In case of the mammalian CTLH complex, so far no alternative SR subunits replacing Gid4 have been identified; however, an alternative Gid4-independent substrate recognition route *via* Wdr26 was reported (21). Interestingly, Ypel5, an additional subunit not present in yeast, binds to the WD40 β-propeller domain of Wdr26 (6), but its role as potential SR or modulator that blocks the Wdr26-binding site needs to be further investigated.

Besides the recruitment of SRs to the GID complex, a distinct oligomeric assembly of the GID complex is crucial for specific substrate ubiquitylation. Structural studies by cryo-EM revealed how the Fbp1 substrate is chelated at the center of the complex by specific interactions with Gid4, thereby facilitating binding and ubiquitylation at multiple sites (6, 46). For the CTLH complex, a closely related oligomeric assembly was described (6, 21), which could again chelate target proteins. In this case, the SR subunit Gid4 is recruited to the complex *via* the C-terminal part of the Armc8α subunit. Together with RanBP9 and Twa1, they comprise the SR scaffolding (SRS) module (Protein Data Bank [PDB] entry: 7NSC). The catalytic RING domain–containing subunits, Rmnd5a and Maea, heterodimerize, and each bind to Twa1 as part of the SRS module. This assembly is further oligomerized *via* dimerization of Wdr26 (supramolecular assembly module, SA) bridging two RanBP9 subunits of different SRS modules, thereby adopting a chelating “ring” shape with space for substrate binding at its center. Noteworthy, also possible SR domains like the WD40 domain of Wdr26 and subunits like Ypel5 face the center of the assembly (6).

In summary, regulation of substrate recognition of the GID–CTLH complex relies both on suitable SRs specifically targeting N-terminal degron sequences paired with an oligomeric assembly, which assists in the positioning and selective targeting of substrates (1). Compared with the yeast complex, the CTLH complex displays a greater variety of interchangeable subunits, thus possibly modulating its oligomeric assembly: Rmnd5b and RanBP10 can replace their paralogs Rmnd5a and RanBP9 (20, 32), Armc8β its longer isoform Armc8α (21), and muskelin, presumably, the more distantly related Wdr26 (Fig. 1*B*). Taken together, this diversity suggests that there is not a single functional CTLH complex with defined subunit composition, instead, multiple different assemblies are possible, and it is expected that these differ in substrate specificity.

To obtain insights into the factors governing CTLH complex assembly, we utilized a toolbox of purified subcomplexes and individual proteins (Fig. 1*C*) to reconstitute different assembly stages *in vitro*. Since knowledge of substrates and their recognition sites within the CTLH complex is still lacking, we focused on the characterization of the scaffold and supramolecular assembly module of the CTLH complex. Facilitated by the availability of pure proteins, we tested putative interactions of subunits with subcomplexes *via* analytical size-exclusion chromatography (aSEC) or native agarose gel electrophoresis (NAGE). In a second step, we determined the oligomeric state of the assembled complex and its single components using multiangle light scattering coupled to aSEC (SEC–MALS). Finally, we quantified the affinities of the binding partners by isothermal titration calorimetry (ITC). We thereby sought to address questions of how and in which oligomeric state Wdr26 and muskelin are integrated into the scaffold of the CTLH complex, whether both subunits bind in a mutually exclusive way and finally, how oligomerization of the Armc8β–RanBP9–Twa1 scaffold module is influenced by its stepwise formation.

## Results

### Modular reconstitution of CTLH complexes

To study the assembly of mammalian CTLH complexes with varying compositions, we generated a toolbox composed of individual proteins and subcomplexes (Fig. 1*C*). The murine variants of Twa1, Wdr26, and Armc8β as well as rat muskelin were recombinantly expressed as single subunits in *Escherichia coli*. Mouse RanBP9, which, when expressed in isolation, was insoluble, could be coexpressed and purified together with Twa1, also from *E. coli*. Similarly, Twa1 was required for the soluble expression of the murine RING subunits Rmnd5a and Maea in insect cells. The CTLH subunits are highly conserved, for example, the pairwise sequence identity of rat and mouse muskelin is 99.6%. Larger CTLH subcomplexes containing the RING subunits, RanBP9, Twa1, Armc8α, and Wdr26 were expressed in insect cells. For both expression systems, the N termini of RanBP9 and Wdr26, which are predicted to be unstructured (52, 53), were deleted to enhance solubility. The following nomenclature for the different protein components in the complexes will be used: muskelin-M, Wdr26-W, RanBP9-R, Twa1-T, Armc8α/β-α/β, and the catalytic Rmnd5a–Maea complex will be abbreviated with cat (as subscript).

### Tight binding to RanBP9 prevents higher order oligomerization of dimeric Wdr26

**Figure 2:**
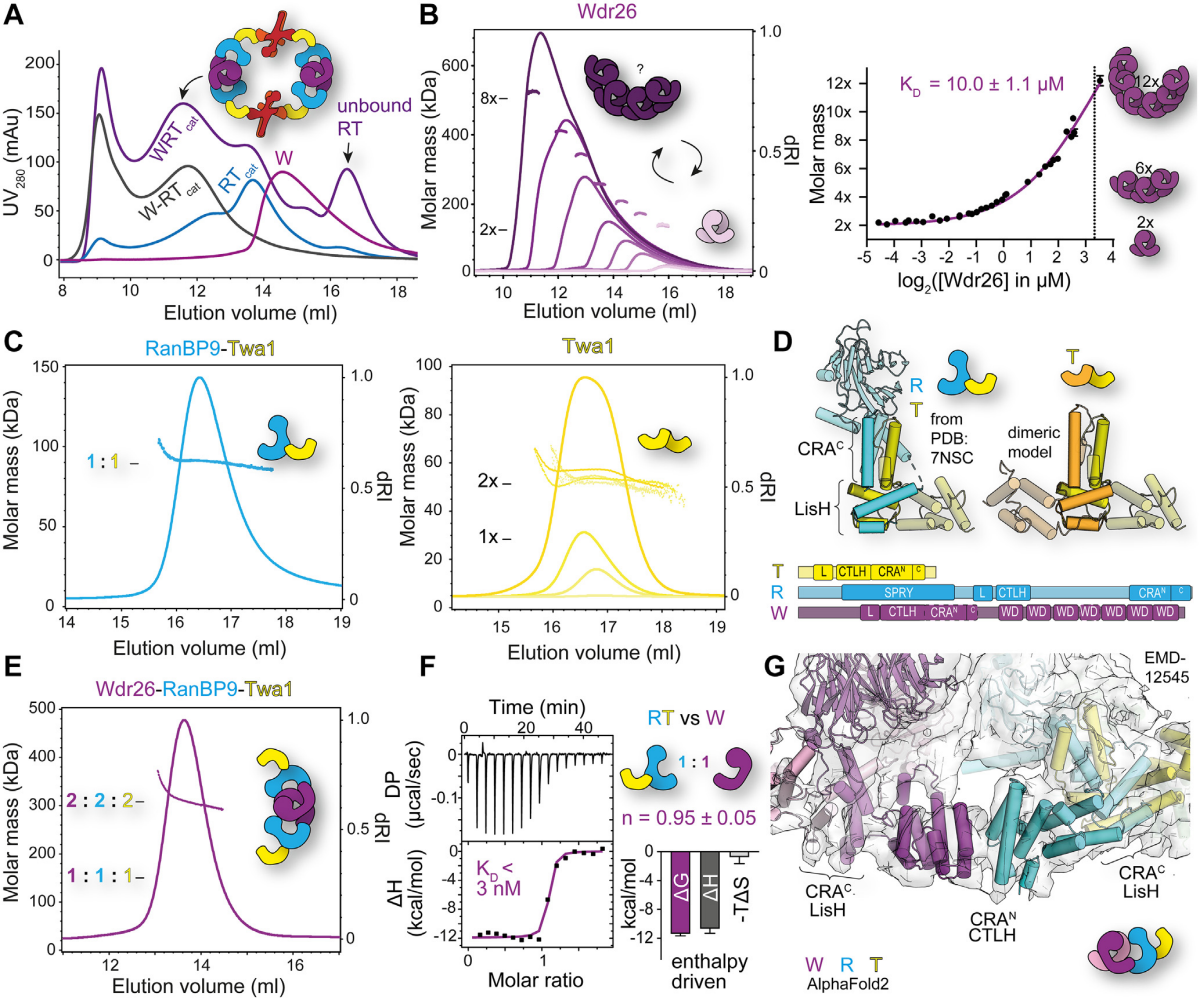
**Tight binding to RanBP9 prevents higher order oligomerization of dimeric Wdr26.***A*, Wdr26-mediated CTLH complex assembly. Analytical SEC documenting assembly of the WRT_cat_ complex from Wdr26 (W) and the coexpressed RT_cat_ complex compared with the coexpressed WRT_cat_ complex. The model indicates the assembly of this complex based on published data (6). See Fig. S1*A* for SDS-PAGE analysis of fractions of the WRT_cat_ elution profile. *B*, self-association of Wdr26 analyzed with SEC–MALS at various protein concentrations (see Fig. S2 for more details). At the elution peak represented by the differential refractive index (dRI) profile signal, molar masses are depicted (*left*). Theoretical molar masses of a dimeric (2×) and octameric (8×) Wdr26 assembly are indicated. The best-fit value of the dissociation constant (*K*_*D*_*)* and its standard error (SE) are reported. *C*, SEC–MALS analysis of the RT complex and Twa1. *D*, based on the cryo-EM structure of RT (derived from PDB entry: 7NSC), a model of a Twa1 homodimer utilizing the same binding mode was constructed by superimposing Twa1 with RanBP9. Schematic representations of the domains of T, R, and W illustrate similarities in their architecture. *E*, molar mass determination of the WRT complex by SEC–MALS. *F*, isothermal titration calorimetry (ITC) studies to determine binding of the RT complex to Wdr26. The differential power (DP) (*upper panel*) was integrated over time, and the released heat (ΔH) plotted against the molar ratio of the RT complex (*lower panel*) and fitted with a one-site binding model. *K*_*D*_ values (*K*_*D*_ = 3.4 ± 3.5 nM) and the signature binding plot were derived from three measurements in which the interaction was not too tight to still permit reliable data analysis of a total of six measurements. The error bars reflect the SE of the change in enthalpy (ΔH), entropy (ΔT), and the Gibbs free energy (ΔG). *G*, model illustrating the Wdr26–RanBP9 interaction. The AlphaFold2 predicted structures of Wdr26, RanBP9, and Twa1 were fitted into the cryo-EM map (EMD-12545) of the human SA module. CTLH, C-terminal to lissencephaly-1 homology motif; L, LisH; MALS, multiangle light scattering; PDB, Protein Data Bank; RT_cat,_ RanBP9–Twa1–Rmnd5a–Maea complex; SEC, size-exclusion chromatography; W, Wdr26; WD, WD40 repeat.

We first focused on the integration of Wdr26 as a subunit that mediates the previously identified supramolecular assembly of the CTLH complex (6, 21). Wdr26 is composed of 625 residues organized into a LisH and CTLH tandem, followed by a CRA-like domain, and C-terminal WD40 repeats folding into a seven-bladed β-propeller domain (Fig. 1, *A*). Adding purified Wdr26 to the coexpressed RanBP9–Twa1–Rmnd5a–Maea (RT_cat_) complex (Fig. S1) led to a large shift in elution volume consistent with further oligomerization of the complex (Fig. 2*A*). This resulted in the same elution profile as a Wdr26-containing complex in which the RT_cat_ module and Wdr26 were coexpressed, thus suggesting that there is no major difference between the complex assembly pathways.

Examination of Wdr26 by SEC–MALS revealed not only the suspected dimer deduced from the existing cryo-EM data (6, 21) but also a large and concentration-dependent shift to lower elution volumes and higher molar masses corresponding to at least an octamer (Fig. 2*B*). This demonstrates that Wdr26, in isolation, has a strong tendency to oligomerize. This oligomerization behavior is dynamic, and the measured molar mass decreases rapidly in the tail of the peak, because of reduced protein concentrations resulting from dilution of the protein during the aSEC run (Fig. S2).

To analyze the self-affinity of the Wdr26 oligomerization, we used the measured molar masses of fixed volume fractions at the elution peaks where the signals were most stable and plotted these against the average peak concentrations of the same interval, which were determined with the refractive index detector (Fig. 2*B*). A one-site binding model was fitted with GraphPad Prism (Dotmatics) to dilution series from four different batches of purified Wdr26. The highest concentration revealed a dodecameric assembly (12-mer), and extrapolation of the unsaturated Wdr26 oligomerization curve suggested a 22-mer (21.7× ± 1.3) at saturation. However, this and the corresponding value of the self-dissociation constant (*K*_*D*_ = 10.0 ± 1.1 μM) should be viewed as estimates since saturating concentrations could not be reached.

To investigate the oligomeric state of the Wdr26-containing CTLH complexes and the Wdr26-binding stoichiometry, we tested its binding to different CTLH subunits (Fig. S3) to identify a simpler system for analysis. Reliable molecular weight determination of the complete Wdr26 CTLH complex assembly with SEC–MALS was impossible because of the large size of this complex, which elutes very close to the void volume of the aSEC column (Fig. 2*A*). Instead, we identified RanBP9 as the only direct binding partner of Wdr26 and used it, in the form of the binary RT complex, to study the assembly process. Wdr26 showed no binding to Twa1 unlike suggested in earlier studies (21). With 228 residues Twa1 is the smallest subunit containing the characteristic LisH–CTLH–CRA domain arrangement (Fig. 1*A*). Its larger relative, RanBP9, comprises 653 residues and possesses, besides larger disordered regions, an additional N-terminal SPRY (SPla and the RYanodine receptor) domain.

Comparative SEC–MALS analyses of the RT complex (Fig. 2*C*) revealed the formation of a heterodimer with an experimental mass of 90.5 ± 2.2 kDa, closely matching the theoretical mass of 91.8 kDa corresponding to a heterodimer of one RanBP9 and one Twa1 molecule. The previously reported stable homodimerization of Twa1 (54), when expressed in isolation, was confirmed by SEC–MALS with molar masses between 51.4 and 56.7 kDa (theoretical dimer molecular weight of 53.6 kDa) at different concentrations (Fig. 2*C*). Formation of the Twa1 homodimer was therefore prevented in favor of the heterodimeric RT assembly. Based on the cryo-EM structure of the human SRS module (PDB entry: 7NSC), RT heterodimerization is mediated by a bipartite interaction mode: LisH dimerization combined with dimerization of the C-terminal helix of the CRA domain (CRA^C^) (Figs. 1*A* and 2*D*). The similar domain architectures of Twa1 and RanBP9 thus indicate that RanBP9 replaces one Twa1 subunit in the Twa1 homodimer, presumably promoted by stronger LisH and CRA^C^ binding.

Further SEC–MALS analysis of the ternary WRT complex (Fig. 2*E*) yielded a molar mass of 312 ± 9 kDa corresponding to a heterohexameric (WRT)_2_ assembly, closely matching the calculated mass of 307 kDa. Since the RT complex exhibited no further oligomerization in solution, dimerization of the ternary WRT assembly must be driven by Wdr26 dimerization. The observed formation of higher oligomeric forms of Wdr26 is apparently prevented when dimeric Wdr26 binds to RanBP9.

Next, we investigated the binding strength of the Wdr26–RanBP9 interaction. ITC studies in which the RT complex was titrated to Wdr26 showed very tight binding and a 1:1 stoichiometry (n = 0.95 ± 0.05). This suggests that each Wdr26 molecule of the dimer binds one RanBP9. Control experiments in which Wdr26 was titrated to Twa1 alone showed no binding, in congruency to the aSEC run in Fig. S3*A* (data not shown). A signature binding plot showed that RanBP9 binding is mostly driven by enthalpic contributions. The derived *K*_*D*_ values were estimated to be around 3 nM but are possibly even smaller, as nanomolar and higher affinities are beyond the limit of what can be accurately measured by ITC (Fig. 2*F*).

Fitting the structures of Wdr26, RanBP9, and Twa1 as predicted with AlphaFold2 (53) into the cryo-EM map of the human SA module (Electron Microscopy Data Bank [EMDB]; EMD-12545) demonstrates that Wdr26 homodimerizes, in a manner analogous to the RT heterodimer, *via* paired LisH–CRA^C^ interactions. The remaining N-terminal part of the CRA domain (CRA^N^) folds back onto the CTLH motif, and both build a functional unit responsible for heterodimerization (Fig. 1*A*). Wdr26 binds to RanBP9 and vice versa *via* CTLH–CRA^N^ dimerization (Fig. 2*G*). The nanomolar affinity of the Wdr26–RanBP9 heterodimerization mediated *via* their CTLH–CRA^N^ domains obviously disrupts higher order oligomerization of Wdr26 with self-affinities in the micromolar range. This indicates that at least parts of the Wdr26 surface are required for higher oligomer formation and are buried when Wdr26 and RanBP9 interact. Further oligomerization of dimeric Wdr26 could therefore be mediated by CTLH–CRA^N^ homodimerization.

### Tight binding to RanBP9 preserves the tetrameric assembly of muskelin

Compared with the yeast GID complex not only Wdr26 but also muskelin displays a similar domain organization as Gid7 (37). The second subunit capable of oligomerizing the CTLH complex as part of the supramolecular assembly module is therefore muskelin (6). It is composed of 735 residues organized into an N-terminal discoidin domain, followed by a LisH and CTLH tandem, six kelch repeats, and a C-terminal module (Figs. 1*A* and S4). In our previous studies investigating muskelin oligomerization, we showed that the protein forms stable tetramers (55). Tetramerization is mediated by two different dimerization interfaces, which can be specifically abrogated by the introduction of three mutations, N114R, F184E, and L196Q, referred to as R, E, or Q variants. The E and Q mutations are located in the LisH motif and prevent LisH-mediated dimerization, whereas the R variant affects the discoidin domain, which disturbs the head to tail dimerization of muskelin (Fig. S4). Using these variants and the wildtype protein, we analyzed the oligomeric state of muskelin within the CTLH complex.

**Figure 3:**
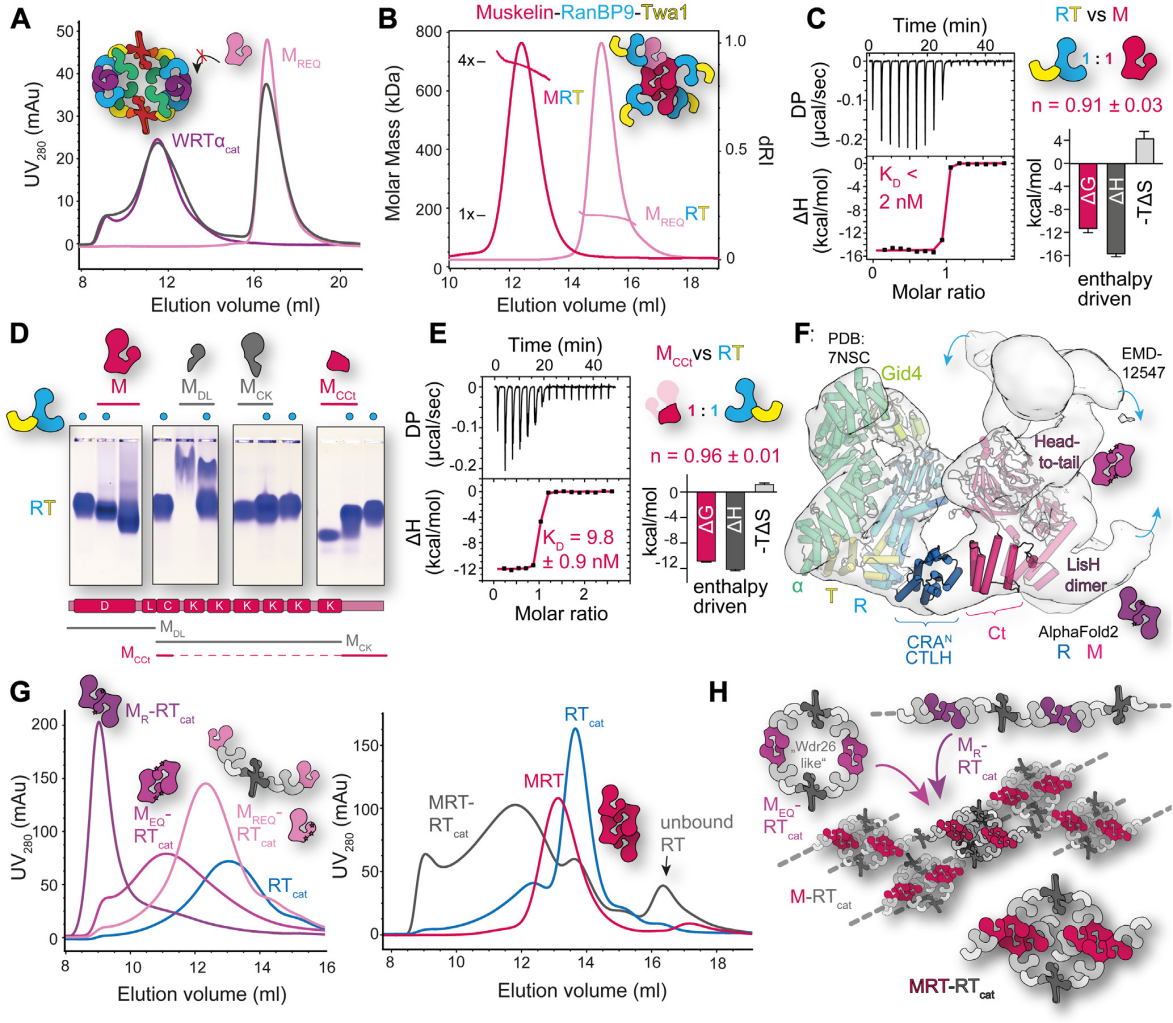
**Tight binding to RanBP9 preserves the tetrameric assembly of muskelin.***A*, mutually exclusive binding of Wdr26 and muskelin to the CTLH complex. UV_280_ elution profiles of an aSEC run documenting the assembly of the WRTα_cat_ complex with the monomeric muskelin mutant M_REQ_ (N144R, F184E, and L196E). For comparison, see Fig. S5. *B*, SEC–MALS analysis of the MRT and M_REQ_RT complexes. *C*, ITC analysis for the binding of RT to muskelin. Similar to Figure 2*F*, the dissociation constant (*K*_*D*_ = 1.7 ± 6.7 nM) and signature binding plot could be derived for only three of six measurements. *D*, native agarose gel electrophoreses (NAGE) of differently truncated muskelin constructs in a complex assembly with the RT complex and as individual proteins. Domains that are included in the construct are depicted as follows: discoidin (D), LisH (L), CTLH (C), Kelch repeats (K), and C-terminal module (Ct). Analysis of further constructs by NAGE are shown in Fig. S6. *E*, ITC study of binding of the RT complex to a M_CCt_ construct comprised of a fusion of the N-terminal part of the CTLH domain with the C-terminal module of muskelin. *F*, model of how the C-terminal module of muskelin binds to RanBP9. The high-resolution cryo-EM structure of RTα–Gid4 (PDB entry: 7NSC) and the AlphaFold2 predictions of muskelin and RanBP9 were fitted into the 10 Å cryo-EM map (EMD-12547). Muskelin dimerization sites are denoted, and blue arrows display further binding sites of RanBP9. *G*, complex assembly of different muskelin variants (M_REQ_, M_R_, and M_EQ_) displayed in shades of purple (*left*) and the MRT complex (*right*) with the RT_cat_ complex as analyzed by aSEC. See Fig. S1*C* for SDS-PAGE analysis of selected fractions. *H*, model illustrating possible assemblies from (*G*). aSEC, analytical size-exclusion chromatography; CTLH, C-terminal to lissencephaly-1 homology motif; ITC, isothermal titration calorimetry; LisH, lissencephaly-1 homology; M, muskelin; SEC-MALS, multiangle light scattering coupled to aSEC; PDB, Protein Data Bank; RT_cat_, RanBP9–Twa1–Rmnd5a–Maea complex; W, Wdr26; α, Armc8α.

In a first step, we determined whether Wdr26 and muskelin bind to the other subunits in a mutually exclusive manner, since they share a similar role in the oligomerization of the complex. Upon addition of the monomeric muskelin variant M_REQ_ to the Wdr26 containing senary (six subunit) WRTα_cat_ complex, no interaction could be detected (Figs. 3*A* and S5), in contrast to the pronounced shift, which was observed by adding M_REQ_ to the quinary RTα_cat_ complex (Fig. S5*A*). Hence, we conclude that once a specific complex has been formed, the respective other protein cannot be added and, on the time scale of our experiments, the subunits are not interchangeable, presumably because of very slow *k*_off_ (dissociation rate constant) rates. Similar to Wdr26, we analyzed complex formation of muskelin with different CTLH subunits and detected binding only to RanBP9 (Fig. S3*B*).

The molar mass of the ternary MRT complex was determined with SEC–MALS to be four times larger (measured: 684 ± 18 kDa, theoretical: 706 kDa) than the mass of the M_REQ_RT complex (measured: 173 ± 4 kDa, theoretical: 177 kDa) (Fig. 3*B*). Thus, tetramerization of the ternary complex is mediated by tetramerization of muskelin. Furthermore, ITC studies exhibited a 1:1 binding of muskelin to RanBP9 with an affinity of <2 nM (Fig. 3*C*), similarly high to that observed for the interaction between Wdr26 and RanBP9 (Fig. 2*F*). This high affinity was observed not only for MRT complex formation but also for the interaction of muskelin with the larger Armc8β containing RTβ complex (Fig. S4*C*).

Next, we analyzed which part of muskelin is responsible for binding to RanBP9. We therefore tested various domain deletion constructs of muskelin for their interaction with the RT complex by NAGE (Figs. 3*D* and S6). Only constructs containing the C-terminal module showed a shift indicative of complex formation. The interaction was confirmed by ITC, in which a fusion construct of the CTLH motif and the C-terminal module of muskelin showed similar binding to RanBP9 as full-length muskelin (Fig. 3*E*). The C-terminal module of muskelin on its own could not be purified, thus preventing further characterization of the interaction. A closer look at the muskelin structure, as predicted by AlphaFold2, revealed that the C-terminal module is not a structural entity on its own but folds back onto the CTLH motif, forming a six-helical bundle with four α-helices being contributed by the C-terminal module (Fig. S6). Fitting the predicted structure of muskelin and the RTα–Gid4 complex (PDB entry: 7NSC) into the 10 Å cryo-EM map (EMDB ID: 12547) generated a model that confirmed that the identified C-terminal module is indeed capable of interacting with RanBP9 (Fig. 3*F*).

Subsequently, we analyzed whether muskelin is still present as a tetramer in the context of the complete CTLH complex. Directly adding tetrameric muskelin to the RT_cat_ complex led to immediate precipitation of both components; hence, complex formation could not be studied. Precipitation was circumvented by protecting the integrity of the muskelin tetramer through preassembly with the RT subunits (MRT), and muskelin binding to the RT_cat_ complex (MRT–RT_cat_) could be observed (Fig. 3*G*). Based on our analysis, at least one copy of RT is exchanged when RT_cat_ is incubated with MRT (Fig. S1*C*). The resulting complex exhibited a similar elution profile as the WRT_cat_ complex (Figs. 2*A*, S1*A* and S5*B*). The preassembly step was not required when muskelin variants impaired in oligomerization were used (M_R_, M_EQ_, and M_REQ_) for complex formation (Fig. 3*G*). A comparison of the elution profiles of the complexes containing the M_R_, M_EQ_, and M_REQ_ variants with the MRT–RT_cat_ complex revealed an almost perfect match for the M_EQ_RT_cat_ complex where dimerization of muskelin *via* the LisH motif is prevented. Complexes with the monomeric M_REQ_ eluted at larger volumes and were therefore smaller, whereas M_R_-containing complexes migrated in the void volume, indicating that they were either aggregated or assembled into higher order species.

A possible model explaining these observations is shown in Figure 3*H*. Since muskelin can interact with four copies of RanBP9, it is likely that also all four binding sites are occupied upon addition of muskelin to the RT_cat_ complex (compare Fig. 3*F*). The heterodimer of Rmnd5a and Maea, however, links, *via* Twa1, two copies of RanBP9, resulting in the formation of an extended sheet-like assembly, which is presumably prone to aggregation. Through preincubation of muskelin with RanBP9, all binding sites are occupied, and only those sites that meet the steric requirements of the RT_cat_ complex are exchanged. This results in a smaller M_EQ_-like dimeric assembly, which in its oligomeric state corresponds to the Wdr26-like assembly. Interestingly, also in the low-resolution MRTα–Gid4 cryo-EM map (EMDB ID: 12547, Fig. 3*F*), only those two muskelin subunits are decorated with RanBP9, which correspond to the M_EQ_ dimer of the tetramer. The model in Figure 3*H* also explains why the inability of the dimeric or monomeric muskelin variants to further oligomerize prevented precipitation.

### Dynamic oligomerization and conformational flexibility of Armc8β

Besides the reciprocal incorporation of Wdr26 and muskelin, another known alternative assembly path of the CTLH complex involves the mutually exclusive incorporation of the Armc8α subunit or its shorter isoform Armc8β (673 *versus* 399 residues) (21). Both subunits share the first 378 residues comprised of seven Armadillo repeat motifs (Fig. 1*A*). Since Armc8α, after expression in insect cells, was prone to aggregation (data not shown), our efforts focused on the characterization of the shorter Armc8β variant.

**Figure 4:**
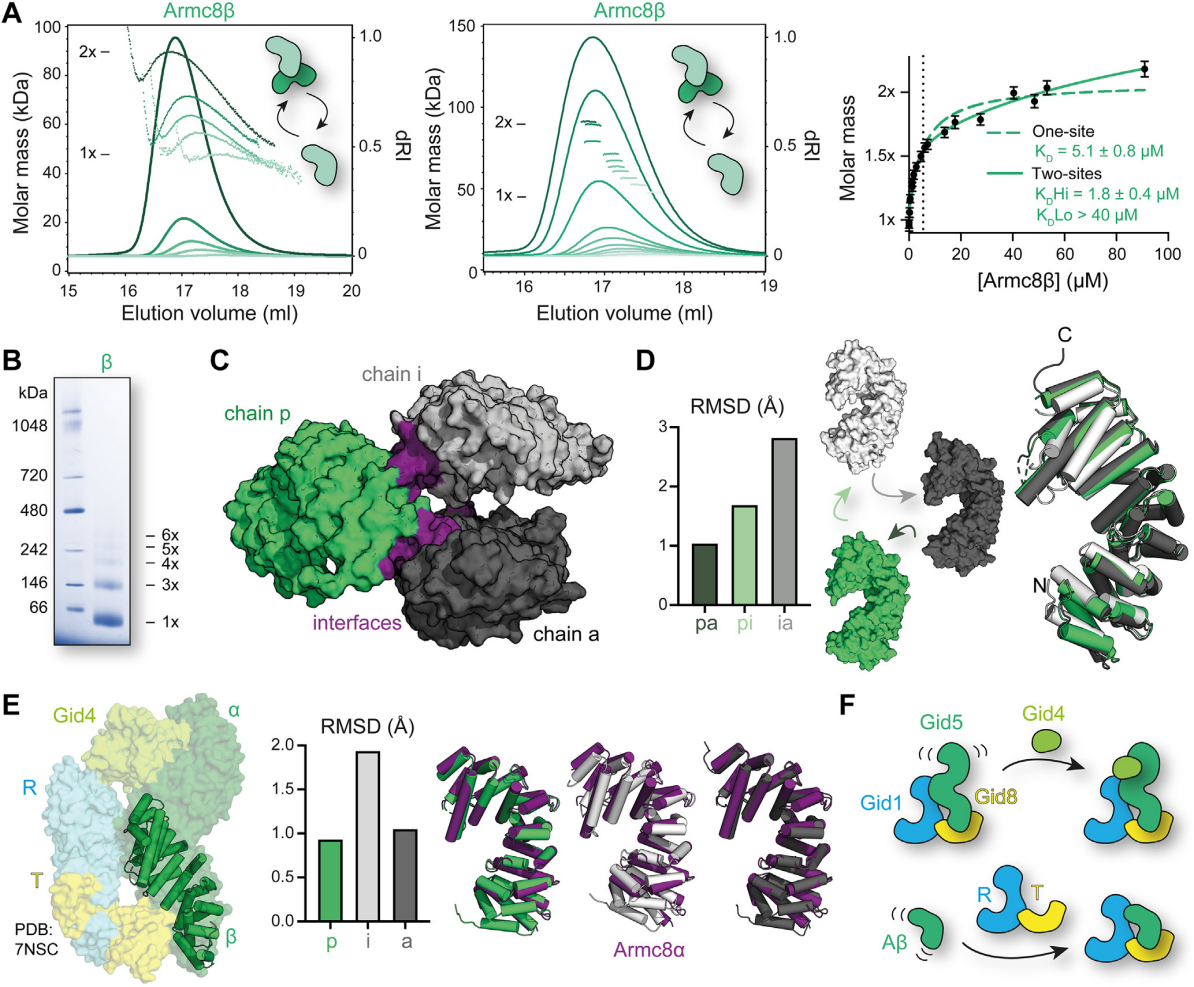
**Dynamic oligomerization and conformational flexibility of Armc8β.***A*, SEC–MALS analysis of the Armc8β self-association (similar to Fig. 2*B*). *B*, blue native PAGE analysis of Armc8β. *C*, the crystal structure of Armc8β (PDB entry: 8A1I) reveals three chains in the asymmetric unit: chains p (green), i (gray), and a (black). *D*, overlay of the three chains in ribbon representation reveals conformational changes. Rms deviations of the different chains toward each other are summarized in the bar chart. *E*, comparison of the p chain (ribbon) of Armc8β with Armc8α as observed in the cryo-EM structure of the RTα–Gid4 complex (PDB entry: 7NSC). Superposition of the three different chains of Armc8β with Armc8α (purple) in ribbon representation illustrates the rms deviations depicted in the bar chart. *F*, model of how complex binding rigidifies the flexibility of Armc8. SEC-MALS, multiangle light scattering coupled to analytical size-exclusion chromatography; PDB, Protein Data Bank.

To determine the oligomeric state of the protein, we carried out a SEC–MALS analysis series at different concentrations (Fig. 4*A*). We observed a concentration-dependent shift toward lower elution volumes, coupled to an increase in the measured molar mass ranging from a monomer to a dimer. Oligomerization is quite dynamic since the molar mass signal drops at the beginning and end of the peak where lower local concentrations of the protein are present, because of dilution on the aSEC column.

To obtain a *K*_*D*_ value of this oligomerization, we plotted the oligomeric state against the concentration, analogous to the analysis of Wdr26 oligomerization. For molar mass and concentration determination, we used a small volume at the peak of the elution where the molar mass signal was highly homogenous. Binding curves were fitted with GraphPad Prism to SEC–MALS measurements from two different protein purifications (Fig. 4*A*). A two-site binding model (*K*_*D*_^high^ = 1.8 ± 0.7 μM, *K*_*D*_^low^ >40 μM) described the data better than a one-site binding model (*K*_*D*_ = 5.1 ± 0.8 μM); however, the *K*_*D*_^low^ could not be accurately determined. This result suggested that dynamic formation of even higher oligomers than homodimers may be possible, which was confirmed by blue native PAGE where a band corresponding to trimeric Armc8β was prominent, and higher oligomers were also visible (Fig. 4*B*).

To identify possible oligomerization sites, we solved the crystal structure of Armc8β after crystallization of selenomethionine-labeled protein by single-wavelength anomalous diffraction at the selenium edge and refined the structure at a resolution of 2.7 Å (Table S1). The crystals contained three chains (denoted as p, i, and a) in the asymmetric unit (Fig. 4*C*). Remarkably, each copy exhibited a distinct conformation (Fig. 4*D*), thus demonstrating dynamic flexibility of Armc8β especially at its termini. The morphed conformations are shown in Movie S1. Chains p and a are more similar as reflected in an rms deviation of 1.04 Å after superimposition of the Cα atoms of all modeled residues, whereas chain i adopts a more compact shape; in this case, the rms deviations are 2.82 Å for the ia pair and 1.69 Å for the ip pair. In terms of oligomerization sites, analysis of the structure with the PISA server (56) detected no significant stable assembly, which is in line with the dynamic nature of Armc8β′s oligomerization behavior. Apparently, the high concentrations in the crystal promoted the observed interactions, nevertheless, since chain p shares an interface with both chain i and chain a, it is tempting to speculate that these two interaction sites could correspond to the two sites identified when analyzing the self-association of Armc8β by SEC–MALS.

To detect structural differences between Armc8β and Armc8α, we compared our Armc8β structures with the structure of Armc8α as determined in the cryo-EM structure of the RTα–Gid4 complex (PDB entry: 7NSC, Fig. 4*E*). Not surprisingly, the overall fold of Armc8α and its shorter isoform Armc8β are very similar. However, in our structure we could model an additional short α-helix at the N terminus. The conformation which Armc8α adopts in the complex is more closely related to the less bent conformation observed for chains a (rms deviation of 0.93 Å) or p (rms deviation of 1.05 Å) of Armc8β, than to chain i (rms deviation of 1.94 Å). This observed structural divergence of both proteins is probably not because of sequence differences. Human and mouse Armc8α are very closely related (overall identity of 98.51% with only two substitutions in the crystallized region), and the C-termianl extension, which is unique to Armc8β, is very flexible and therefore could only be partially resolved (5–9 residues). It is more likely that structural differences arise because Armc8α is being present in a complex, whereas Armc8β was analyzed in isolation. Interactions with RT and Gid4 restrict the flexibility of Armc8α and lock it in a conformation capable of complex binding.

A similar principle becomes apparent when comparing the cryo-EM structure of the yeast Armc8α homolog Gid5 in the substrate–receptor-unbound state with the bound state (46). Gid5, in complex with the Gid1–Gid8 (RT) scaffold, shows great flexibility in its free C-terminal SR binding half. Upon binding of the SR Gid4, this flexibility is lost and Gid5 is locked in a distinct conformation (Fig. 4*F*). In analogy to the rigidification of the flexible C-terminal part of Gid5–Armc8α after SR recruitment, one could envision how binding partners like RanBP9 and Twa1 restrict the overall flexibility of Armc8β.

### Tight binding to RanBP9 prevents higher order oligomerization of the Twa1–Armc8β complex

**Figure 5:**
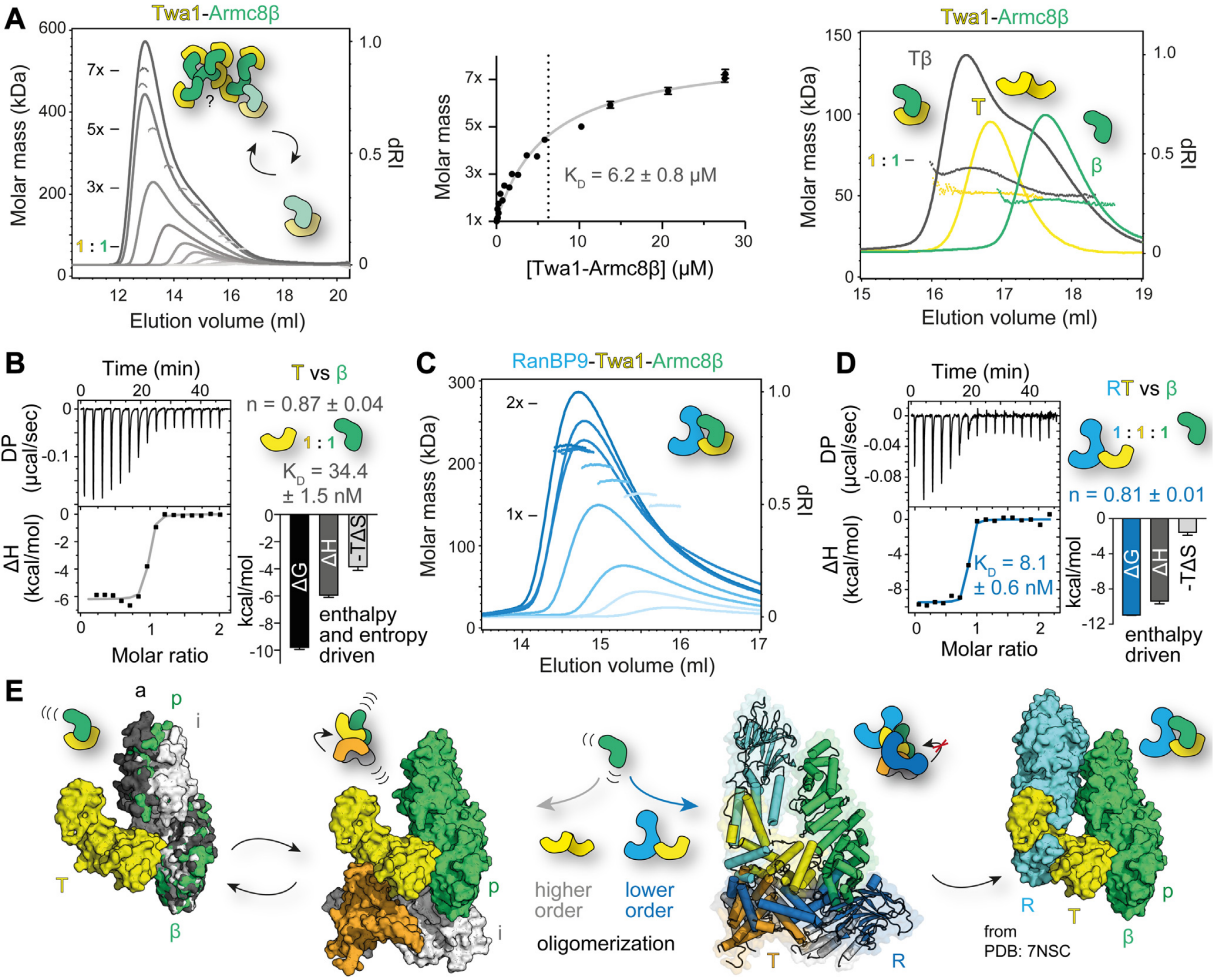
**Tight binding to RanBP9 prevents higher order oligomerization of the Twa1–Armc8β complex.***A*, SEC–MALS analysis of the Tβ complex self-association conducted as in Figures 2*B* and 4*A*. Full molar mass peak profiles are shown in Fig. S1*B*. In addition, assembly and molecular weight analysis of the binary complex at low concentrations are depicted (*right*). *B*, ITC binding studies of Twa1 to Armc8β displayed as for Figure 3*C*. The affinity constant *K*_*D*_ and signature binding plot parameters including their standard errors were derived from 18 measurements. *C*, molecular weight determination of RTβ complex self-association with SEC–MALS at different concentrations was limited by the instability of the complex at higher concentrations. See Fig. S1*C* for full molar mass profiles. *D*, ITC analysis of the RT complex binding to Aβ derived from seven measurements. *E*, model of how the oligomerization of the binary Tβ complex is prevented upon addition of RanBP9. To demonstrate this the Twa1-Armc8α module from the RTα–Gid4 complex (PDB entry: 7NSC) was superimposed with the three Armc8β conformations observed in the crystal structure. ITC, isothermal titration calorimetry; SEC-MALS, multiangle light scattering coupled to analytical size exclusion chromatography; PDB, Protein Data Bank.

Interestingly, the flexibility and oligomerization tendency of Armc8β was not weakened upon binding to Twa1. On the contrary, Twa1 and Armc8β dynamically formed large oligomeric Tβ complexes with a size of up to 520 kDa (Figs. 5*A* and S1*B*). To exclude contributions from dynamic Armc8β oligomerization, Tβ complex formation was tested at low concentrations (Fig. 5*A*) where Armc8β was almost completely present as a monomer (measured: 47.2 ± 1.1 kDa, theoretical: 44.8 kDa) and Twa1 as a dimer (measured: 51.7 ± 1.7 kDa, theoretical: 53.6 kDa). The measured molar mass of the Tβ complex was in line with a 1:1 complex (measured: 66.6 ± 1.8 kDa, theoretical: 70.8 kDa). The slightly lower measured mass compared with the theoretical value is explainable by the coelution of unbound protein that influences the laser scattering to lower masses. Binding of Armc8β to Twa1 disrupts, similar to RanBP9 binding, Twa1 homodimerization. However, how this is mechanistically achieved is not as easily conceivable as for RanBP9 binding, since homodimerization of Twa1 *via* LisH–CRA^C^ should not interfere with the Armc8β-binding site (Fig. S1*B*).

As observed for the Armc8β oligomerization (Fig. 4*A*), oligomerization of the Tβ heterodimer was very dynamic. The molar mass profiles (Fig. S1*B*) decreased substantially toward the ends of the elution peaks where lower protein concentrations prevail. Likewise, plotting the concentration-dependent shift in molar masses measured for the peak fractions yielded a self-binding curve, which could be sufficiently described with a one-site binding model. The affinity of this higher order oligomerization was determined to be in the low micromolar range (*K*_*D*_ = 6.2 ± 0.8 μM).

This result already indicated that the affinity of both proteins toward each other must be even higher. Indeed, ITC studies of Twa1 binding to Armc8β (Fig. 5*B*) showed a much tighter affinity of 34.4 ± 1.5 nM. A thermodynamic analysis of the data revealed that the interaction was enthalpically and entropically driven. The increase in entropy could possibly result from disruption of the Twa1 dimer, which is expected to expose hydrophobic residues buried in the interface. At the same time, our SEC–MALS data indicated disruption and subsequent progressive oligomerization of the complex at increasing concentrations up to a (Tβ)_8_ heterohexadecameric assembly (8.2× ± 0.3).

In contrast, SEC–MALS analysis of the ternary RanBP9–Twa1–Armc8β (RTβ) complex only showed a very weak propensity for oligomerization, reaching at the highest concentration a mass (measured: 220 ± 5 kDa) which was in between an RTβ heterotrimeric (theoretical: 137 kDa) and an (RTβ)_2_ heterohexameric assembly (theoretical: 273 kDa) (Figs. 5*C* and S1*C*). These measurements were, however, limited by the concentration range, as higher concentrations led to precipitation of the complex. Nevertheless, the binding affinity of RT to Armc8β could be determined by ITC, yielding a dissociation constant of 8.1 ± 0.6 nM, which corresponds to an approximately fourfold tighter binding than in the binary Tβ complex (Fig. 5*D*). Furthermore, in the ternary complex, the entropic contribution was substantially reduced, presumably because the hydrophobic residues exposed upon dissociation in the Twa1 dimer remain buried in the RanBP9–Twa1 interface. At the same time, the enthalpic contribution is more favorable, which may be explained by the additional binding site formed between Armc8β and the SPRY domain of RanBP9 and the ensuing α-helix.

A possible model to explain how the additional binding of RanBP9 to the Tβ complex can prevent its higher oligomerization is displayed in Figure 5*E*. Two concepts are illustrated: restricting Armc8β′s flexibility and increasing the steric hindrance upon additional RanBP9 binding. Overlaying the three conformations of the Armc8β structure to the binding site of Armc8α with Twa1 (PDB entry: 7NSC) shows that there is sufficient space for Armc8β to adopt its different conformations and therefore to remain flexible in the Tβ complex. However, upon RanBP9 binding, Armc8β′s flexibility is restricted and, similar to how Armc8α is integrated into the complex, the p-conformation is locked when present in the complex. Oligomerization sites that promoted the higher order oligomerization of the Tβ complex become either occupied through protein–protein interactions like RanBP9 binding or are blocked by steric hindrance. Nevertheless, how the higher order oligomerization of the Tβ complex is achieved mechanistically remains elusive. Twa1 stably dimerizes even at concentrations where the binary Tβ complex forms higher order oligomers (Fig. 2*C*). At the same time, the dynamic oligomerization of Armc8β is mostly restricted to dimers although higher oligomers were detectable (Fig. 4*A*). Hence, binding of Armc8β toward Twa1 must unlock additional stronger oligomerization sites, possibly *via* disruption of Twa1 dimerization, that are either novel and/or enhance existing oligomerization sites. Overlaying Twa1 *via* the aligned binding site of Armc8α toward the p and i conformations of the Armc8β structure show that Twa1 might interact with itself, which could thereby enhance dynamic Armc8β oligomerization (Fig. 5*E*).

## Discussion

**Figure 6:**
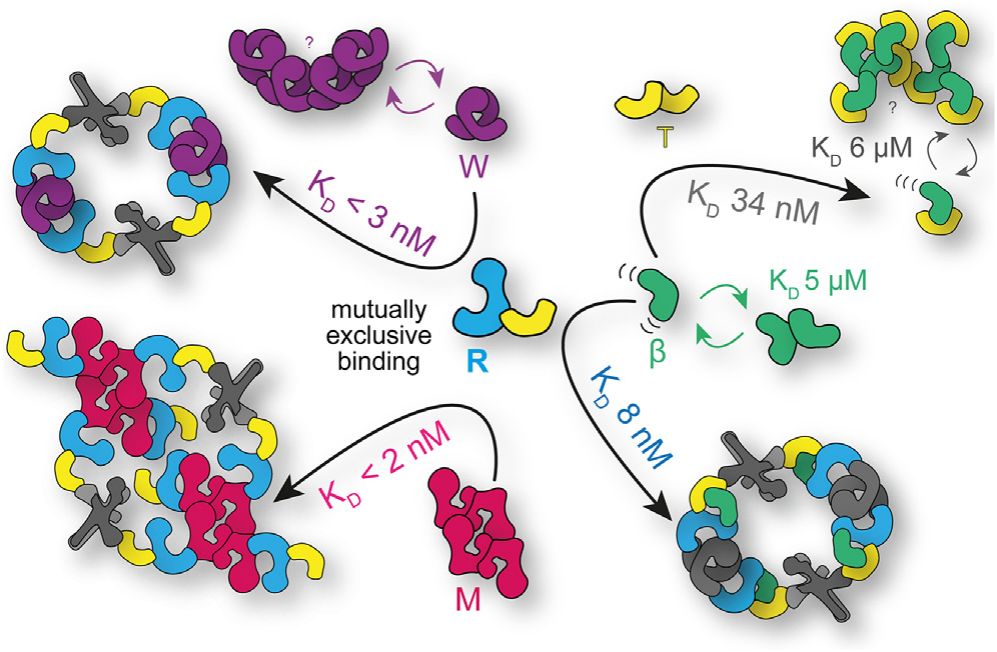
**Tight binding to RanBP9 governs differential oligomerization of CTLH complex assemblies.** CTLH, C-terminal to lissencephaly-1 homology motif; M, muskelin; R, RanBP9; T, Twa1; W, Wdr26; β, Armc8β.

The modular architecture of the CTLH complex is presumably key to the functional diversity of this E3 ligase. Hence, it is important to investigate the assembly pathways leading to different subunit compositions of the CTLH complex. By using recombinantly expressed subunits or smaller modules of the CTLH complex, we studied its assembly by biochemical and biophysical techniques. These studies revealed the tendency of several subunits to oligomerize into higher order structures, which presumably represent nonproductive assembly intermediates. This tendency prevented the respective partner proteins to be present, which instead promote heterologous interactions. Our studies revealed that RanBP9 is perfectly suited because of its position at the heart of the complex, to regulate differential oligomerization of CTLH complex assemblies. With binding sites for Twa1, Armc8β, Wdr26, and muskelin exhibiting high affinities in the (sub-)nanomolar range (Fig. 6), RanBP9 binding alters the oligomeric state of assemblies by either preventing or promoting oligomerization through interaction with its LisH–CRA^C^ or CTLH–CRA^N^ domain.

The RanBP9–Twa1 interaction represents an example where RanBP9 prevents oligomerization. Here, LisH–CRA^C^-mediated homodimerization of Twa1 is prevented by binding to the respective LisH–CRA^C^ domain of RanBP9, thereby forming RanBP9–Twa1 heterodimers (Fig. 2, *C* and *D*). In general, LisH–CRA^C^-mediated dimerization plays an important role in the assembly of the core CTLH complex. Besides Twa1, also Wdr26 dimerization is LisH–CRA^C^mediated, and binding of the RT complex does not disrupt this dimerization (Fig. 2). Whether this is the case because of a stronger LisH–CRA^C^ homodimerization of Wdr26 than a LisH–CRA^C^ heterodimerization with RanBP9 or Twa1 or a kinetic effect with a slow *k*_off_ value is not yet clear. Rmnd5a–Maea heterodimerization is, based on the homologous Gid2–Gid8 structure (6), established *via* LisH–CRA^C^ interactions and facilitated by the RING domains and N-terminal coiled-coil regions present in both subunits. Binding of other LisH–CRA^C^-containing subunits does not disrupt the heterodimer. Therefore, it is explainable why subunits that heterodimerize like RanBP9 or Rmnd5a–Maea need their respective binding partner for reciprocal stabilization and increased solubility (Fig. 1).

Homodimeric or heterodimeric modules are further connected by specific CTLH–CRA^N^ domain interactions. Twa1 binds to each of the Rmnd5a–Maea CTLH–CRA^N^ domains, thereby leading to higher order oligomerization, possibly by alternating Rmnd5a–Maea heterodimerization and Twa1 homodimerization (Figs. S1*B* and S3). Twa1, however, does not bind to muskelin or Wdr26 (Fig. S3), instead the CTLH–CRA^N^ domain RanBP9 is an important determinant that recruits either Wdr26 or muskelin to the complex with similar (sub-)nanomolar affinities (Fig. 6). Upon binding of Wdr26 with its CTLH–CRA^N^ domains to RanBP9, higher order oligomerization of Wdr26 is disrupted (Fig. 2). Since muskelin lacks a CRA domain, its C-terminal module is responsible for RanBP9 binding and folds, similar to a CRA^N^ domain, back to the CTLH motif (Fig. 3). Once bound, the subunits are not interchangeable and enable stable differential assemblies (Figs. 3*A* and S4).

Since tetramerization of muskelin is maintained in the CTLH complex, it may function as a branch point and allow multiple higher order oligomeric assemblies that facilitate simultaneous chelation of more than one substrate molecule. Recently, a cryo-EM structure revealed a 5 MDa large cage-like assembly comprised of three chelator–GID complexes (60 subunits in total) that are connected by additional Gid7 homodimerization besides LisH–CRA^C^ homodimerization and CTLH–CRA^N^ binding to Gid1 (RanBP9). This additional homodimerization is mediated *via* a differential CTLH–CRA^N^ dimerization site (51). Our studies of the WRT complex do not support a further oligomerization of Wdr26 beyond a dimeric state. However, muskelin being present as a tetramer in the MRT complex could readily mediate those interactions to form an analogous cage-like CTLH complex. Interestingly, we identified smaller peaks, close to the void volume of the size-exclusion column, in all our assemblies of the CTLH complex containing the catalytic module (Figs. 2*A*, 3*G*, S1, S3 and S5), thus suggesting that large assemblies in the mega Dalton range are formed.

The discovery that the CTLH complex targets muskelin for degradation (57) suggests that the complex might regulate its clustering abilities and reciprocal Wdr26 incorporation. In *Drosophila* embryos, temporal degradation of muskelin enables substrate ubiquitylation in a Gid4-independent manner (25, 26). Whether this is an effect based on differential oligomerization or restricting access to substrate adaptors remains to be shown. In mammals, however, Wdr26, which harbors a WD40 repeat domain, was identified as a substrate adaptor that is important for Gid4-independent degradation of the transcription factor Hbp1 (21). Interestingly, the WD40 repeat domain is known to be present in several substrate adaptors in SCF ubiquitin ligase complexes (58).

We speculate that muskelin and Wdr26 function not only as alternative oligomerization modules but also are important as potential SR modules—either *via* direct substrate binding to the kelch (muskelin) or WD40 repeat domains (Wdr26) or *via* recruitment of additional receptors or modulators, for example, Ypel5 to Wdr26 (6), which, in turn, could also influence complex oligomerization. With respect to muskelin, its subcellular localization depends on its oligomeric state (55), and it remains an open question how regulation of the oligomeric state of muskelin influences CTLH complex localization and function. Although the RanBP9 binding and tetramerization sites are independent of each other, the order of binding to the CTLH complex and the oligomeric state of muskelin strongly influence the stability of the assembly; however, the interplay between these factors is not yet entirely clear (Fig. 3).

Binding and oligomerization of the CTLH complex by subunits of the supramolecular assembly module is independent of the recruitment of Armc8α to the complex (Figs. 2*A*, 3*G* and S4*C*). However, incorporation of Armc8α or its shorter isoform Armc8β is crucial to regulate substrate binding *via* the Gid4 route. Reflected by its domain architecture lacking the LisH–CTLH–CRA domains, Armc8α as an SR adaptor is dispensable to establish the overall scaffold of the CTLH core complex. This subunit is also not as stably integrated into the complex as the other subunits and was lost in initial purifications of the RTα_cat_ and WRTα_cat_ complexes.

Our structural studies of isolated Armc8β showed significant conformational flexibility and hence dynamic self-association, which was even enhanced upon Twa1 binding (Fig. 5). Self-dissociation constants in the low micromolar range were determined for Armc8β, the Tβ complex, and estimated for Wdr26 and the RTβ complex (Figs. 2*B*, 4*A*, 5*A* and S1*C*). Whether this self-association is relevant at the local concentrations in the cell remains to be investigated; however, we suspect that it may nucleate the complex and assist in the formation of higher order assemblies. Tighter binding in the low nanomolar range to RanBP9–Twa1, however, prevented formation of higher order oligomers (Fig. 6) and stabilized the heterotrimeric assembly of the RTβ scaffolding module and a stable heterohexameric WRT supramolecular assembly. Consequently, RanBP9 binding to a combination of defined oligomers and dynamic self-associated subunits orchestrates differential oligomerization of the CTLH complex.

It will be interesting to investigate the role of RanBP10, which, as a close paralog, may replace the organizing role of RanBP9, for example, as already demonstrated in stage-dependent assemblies important for erythropoiesis (32) and thereby dictate the ability of the CTLH complex to bind to distinct substrates. Whether this modulation of function is based on its scaffolding function or occurs *via* direct substrate interactions remains to be determined. With respect to substrate binding, it can be readily envisioned how the SPRY domain of either RanBP9 or RanBP10, which not only faces the center of the “ring-like” chelate assembly but is stabilized in its position through the interaction with Armc8β, could serve as a possible substrate-recruiting domain. While current efforts focus only on the exploitation of Gid4 to bridge proteins of interest with proteolysis targeting chimeras to the E3 ligase for targeted protein degradation (59), the CTLH complex offers with its possible substrate adaptor domains multiple other ways beside Gid4 to target substrates. Future studies are required to uncover the full therapeutic potential of the CTLH complex.

Taken together, it becomes evident that the unique architectures of the CTLH complex subunits entails the potential to arrange themselves in a multitude of differential functional assemblies. While the catalytic activity of the different CTLH complexes will be ensured by the presence of the RING subunits Rmnd5a and Maea, there is, even at this level, the potential to modulate the properties of the CTLH complex by replacing Rmnd5a with its counterpart Rmnd5b. As discussed previously, this heterogeneity appears to be a recurring theme for the CTLH family, and our study provides initial insights into this functional diversity by defining alternative assembly pathways of the CTLH complex. Conformational flexibility, higher order oligomerization of single subunits and small complexes, sequential binding orders, tight binding of mutually exclusive subunits, and clustering of higher order assemblies are factors that govern CTLH complex assembly and thereby its function. Future studies are required to investigate the regulation of the assembly to fully understand the determinants of specific substrate targeting of the CTLH complex E3 ligase platform.

## Experimental procedures

### Protein expression and purification

Wdr26 expression as 6x histidine (His_6_)-tagged fusion protein and His_6_-small ubiquitin modifier (SUMO)–tagged expression of RanBP9 of the RT complex, Twa1, Armc8β, and muskelin variants were performed in *E. coli* and used for an initial nickel-affinity capture step. Larger catalytic module–containing assemblies (T_cat_, RT_cat_, RTα_cat_, and WRTα_cat_ complex) were baculovirally expressed in Hi5 insect cells with affinity tags on Maea (His_6_-TwinStrep), Twa1 (His_6_), and RanBP9 (3xFLAG), which were similarly used for an initial nickel affinity chromatography step. After tag removal of the bacterially expressed subunits, an optional anion exchange chromatography step was followed, and a final SEC step was employed for all samples. Detailed experimental conditions during expression and purification as well as cloning procedures of the expression plasmids are explained in the supporting information section.

### SEC–MALS

To determine the oligomeric state of CTLH subunits and subcomplexes, a Superose 6 10/300 GL (Cytiva) size-exclusion column attached to an Äkta Purifier 10 system coupled in line to a DAWN HELEOS 8+ light scattering detector and an Optilab T-rEX refractive index detector (both from Wyatt technology) was used. About 100 μl of protein sample with varying concentrations were injected on a column, freshly equilibrated with SEC buffer, and separated while recording the dRI and laser scattering signals. Data were analyzed and plotted using the Astra 6.1.5 software (Wyatt). To determine the dissociation constant *K*_*D*_ describing the self-association of proteins, the oligomeric state (molar mass) was plotted against the concentration as determined with the dRI detector at an elution peak fraction, which was chosen so that stable molar masses were measured. The fraction sizes were 0.4 ml for Wdr26 and the ternary RTβ complex, 0.3 ml for the binary Tβ complex, and 0.2 ml for Armc8β. One-site and two-site binding curves were fitted to dilution series data from at least two different purifications with GraphPad Prism 9.

### ITC

Proteins were dialyzed overnight into ITC buffer (20 mM tricine [pH 8.0], 150 mM or 200 mM NaCl, and 1 mM Tris(2-carboxyethyl)phosphine). After centrifugation, protein concentrations were measured using the absorption at 280 nm and the respective calculated molar extinction coefficients and diluted to concentrations between 50 and 150 μM when present in the syringe and 10 times lower when present in the cell. ITC measurements were performed using an ITC-200 instrument (Malvern Analytics) at 25 °C. Proteins were titrated in 16 injections with the first containing 1.25 μl and the remaining 2.5 μl to their putative binding partners. Titrations were repeated with different protein purification batches. Data were analyzed using a one-site binding model with the Microcal software (Malvern Analytics) in Origin.

### Further biochemical analyses

Details of the aSEC for interaction studies, NAGE, and blue native polyacrylamide gel electrophoresis are described in the supporting information section.

### Crystallization, data collection, and structure solution of Armc8β

Crystals of selenomethionine-derivatized Armc8β were obtained using a hanging drop vapor diffusion setup. Diffraction data were collected at the European Synchrotron Radiation Facility, and the structure was solved using single-wavelength anomalous diffraction method. Experimental details on crystallization conditions, data collection, experimental phasing, model building, and refinement are provided in the supporting information section.

## Data availability

The atomic coordinates and crystallographic structure factors for Armc8β have been deposited in the PDB (https://www.ebi.ac.uk/pdbe/) with accession code 8A1I.

## Supporting information

This article contains supporting information.

## Conflict of interest

The authors declare that they have no conflicts of interest with the contents of this article.
