# Supplementary material for: RanBP9 controls the oligomeric state of CTLH complex assemblies
Source: J Biol Chem. 2023 Jan 5;299(2):102869. doi: 10.1016/j.jbc.2023.102869 (PMC9932110; doi:10.1016/j.jbc.2023.102869)
Supplement: Supplemental information [file mmc2.pdf]

Supporting Information for

RanBP9 controls the oligomeric state of CTLH complex assemblies

Pia Maria van gen Hassend[1], Aparna Pottikkadavath[1], Carolyn Delto[1], Monika Kuhn[1], Michelle Endres[1], Lars Schönemann[1] and Hermann Schindelin[1]

[1]Julius-Maximilians-Universität Würzburg, Rudolf Virchow Center for Integrative and Translational Bioimaging, Institute of Structural Biology, Josef-Schneider-Str. 2, 97080 Würzburg, Germany.

Hermann Schindelin

Email: hermann.schindelin@virchow.uni-wuerzburg.de

Cloning and plasmid construction

Cloning of muskelin constructs for bacterial expression and the generation of the pETM-SUMO vector which harbors an N-terminal 6x histidine-small ubiquitin modifier (His_6-SUMO) tag were described earlier (1). Constructs of murine Rmnd5a, Maea, RanBP9, Twa1, Armc8α and Wdr26, were amplified by PCR from cDNA obtained from codon-optimized synthetic DNA (Invitrogen™, Life Technologies) for Maea, Twa1, Armc8α and Wdr26, from the Rmnd5a-pEGFP-C1 plasmid (2) (the human homolog is identical to murine), kindly provided by Thorsten Pfirrmann, and from the RanBP9-pcDNA3.1(+) plasmid, kindly provided by Mary Ellen Palko, as templates.

For bacterial expression, Wdr26 was subcloned with an N-terminal deletion (residues 1-101) of the flexible part as a NcoI/NotI fragment in the pETM-11 vector, carrying an N-terminal His_6-tag followed by a TEV cleavage site. Similarly, full-length Twa1 was inserted as an NcoI/NotI fragment behind the His_6-SUMO tag of the pETM-SUMO vector and as an NdeI/XhoI fragment into the second multiple cloning site of the pCDF-Duet1 vector. N-terminally truncated (residues 1-68) RanBP9 was subcloned into the pETM-SUMO vector using the SLIC method (3). For generation of the shorter Armc8β isoform, the C-terminal 21 specific amino acids were added after residue 380 of Armc8α via amplification with PCR and subcloned as an NcoI/XhoI fragment into the pETM-SUMO vector.

For baculovirus generation for insect cell expression with the MultiBac system (4, 5), a combination of vectors were used: the pFastBac™Dual (pFBD) vector (Bac-to-Bac Invitrogen™, Life Technologies) and pFBDM for integration via Tn7 transposition and the pSPL-vector using Cre-loxP site-specific recombination. For Rmnd5a-Maea-Twa1 (T_{cat}) complex expression, Maea was subcloned as a BamHI/NotI fragment into the pOpiE2 vector (6) after the His_6-TwinStrep-3C-tag. In a second step, tagged Maea was further subcloned as an NheI/NotI fragment into the pFBD vector, followed by Rmnd5a as an Nco/NheI fragment. Twa1 was subcloned as an NcoI/NotI fragment behind the His_6-TEV-tag of the pETM-11 vector and, as tagged version, into the pSPL vector using SLIC. For additional RanBP9 expression in the RT_{cat} complex, an N-terminal 3xFLAG-tagged was introduced via PCR to RanBP9 and subcloned using SLIC into the Twa1-pSPL vector. For additional Armc8α and Wdr26 expression into the $RT\alpha_{cat}$ and $WRT\alpha_{cat}$ complex, Armc8α and Wdr26 were subcloned into the pFBDM vector using SLIC, followed by transfer of the Rmnd5a-Maea cassette of the pFBD vector into pFBDM via SLIC.

Bacterial expression of CTLH subcomplexes and isolated subunits

Expressions of Armc8β, Twa1, muskelin variants, RanBP9-Twa1 and Wdr26 were performed in BL21 CodonPlus® (DE3) RIL *E. coli* cells using LB medium supplemented with 34 µg/ml chloramphenicol and 50 µg/ml kanamycin. In case of RanBP9-Twa1 co-expression, 50 µg/ml streptomycin was additionally added. Cells were grown at 37 °C to an optical density (OD_{600}) of 0.6-1.2 and protein expression was induced with 0.5 mM isopropyl ß-D-1-thiogalactopyranoside (IPTG) at 20°C or 18°C for Wdr26. To generate selenomethionine (SeMet) labeled Armc8β (SeMet-Armc8β) cells were grown in M9 medium (8 g/l Na_2HPO_4, 4 g/l KH_2PO_4, 0.5 g/l NaCl, 0.5 g/l NH_4Cl, 0.4% (w/v) glucose, 1 mM $MgSO_4$, 0.3 mM $CaCl_2$, 1 mg/l biotin, 1 mg/l thiamin, 25 mg/l EDTA, 4.15 mg/ml $FeCl_3 \cdot 6\ H_2O$, 0.42 mg/l $ZnCl_2$, 65 µg/l $CuCl_2 \cdot 2\ H_2O$, 50 µg/l $CoCl_2 \cdot 6\ H_2O$, 50 µg/l H_3BO_3, 8 µg/l $MnCl_2 \cdot 6\ H_2O$) to an OD_{600} of 0.5 at 37 °C. The medium was supplemented with 100 mg/l lysine, phenylalanine, and threonine, 50 mg/l isoleucine, leucine, and valine and 70 mg/l selenomethionine, and expression of the protein was induced after 15 min. Cells were harvested by centrifugation for 10-20 min at 6,000 x g after 20-22 h of expression time and flash-frozen in liquid nitrogen and stored at -80 °C or directly used for purification.

Insect cell expression of CTLH complexes containing the catalytic module

Insect cells were cultured in either Ex-Cell 420 serum-free medium-SFM (Sf21 cells for baculovirus generation) or Ex-Cell 405 serum-free medium (Hi5 cells for expression) at 27.5 °C. Cell densities were kept between 0.5 and $5x10^6$ cells/ml. To prepare the initial baculovirus titer, V_0 (concentration of infectious particles in the virus stock), $1x10^6$ Sf21 insect cells were seeded into single wells of a 6-well plate in 2 ml Ex-Cell 420 SFM. After 30 min the medium was removed, and cells were transfected using SuperFect transfection reagent (Qiagen). After transfection, cells were overlaid with 3 ml Ex-Cell 420 SFM and the 6-well plate was sealed, and after 4 days of incubation, the supernatant (V_0) was taken off and stored at 4°C until further use. To prepare a V_1 baculovirus titer, a culture of 20 ml Sf21 cells at $0.7x10^6$ cells/ml was infected with 10% (v/v; 2 ml) V_0 initial baculovirus titer and incubated for 4 to 6 days. For expression in Hi5 cells, V_1 baculovirus was added to a final concentration of 10% (v/v) with a density of $1.5x10^6$ cells/ml. After 72 h expression time, cells were harvested at 200 x g for 5 min.

Purification of CTLH complexes, subcomplexes and isolated subunits

For purification, initial Ni-affinity capture (Immobilized Metal Affinity Chromatography, IMAC) was followed by optional anion exchange chromatography (AIEX) and finalized by size exclusion chromatography (SEC). For lysis, cells were resuspended in a suitable LEW buffer (lysis-equilibration-wash) supplemented with DNaseI (AppliChem), EDTA-free cOmplete protease inhibitor cocktail (Roche) and lysozyme (Carl Roth) for bacterial cells or 0.2% (v/v) NP-40 for insect cells. *E. coli* cells were ruptured in two passages through a microfluiduzer (Microfluidics) at 1.5 kbar, and the lysate was cleared by centrifugation at 38,000 x g for at least 30 min. Insect cells were sonicated with an amplitude of 100% with a pulse 1 s on / and 2 s off for five times, and the lysate was cleared by ultracentrifugation at 235,000 x g for 1.5 h.

For purification of Armc8β and its SeMet-derivative as His_6-SUMO fusion proteins, the cleared supernatant was loaded on a gravity-flow Protino® Ni-IDA column (Macherey-Nagel) pre-equilibrated with LEW I buffer (50 mM tricine pH 8.0, 500 mM NaCl, and 1 mM TCEP) and incubated for 1 h while gently shaking. After washing with 15 column volumes (CV) of LEW I buffer, on-column overnight cleavage of the His_6-SUMO tag with the SUMO specific protease dtUD1 was performed in one additional CV of LEW I buffer. Cleaved protein was eluted with 4 CV of LEW I buffer and fractions containing Armc8β were combined, concentrated using 30 kDa cutoff Amicon spin concentrators, centrifuged for at least 20 min at 30,000 x g, and used for SEC utilizing a HighLoad™ 26/600 Superdex™ 200 pg column (Cytiva) pre-equilibrated in SEC I buffer (20 mM tricine pH 8.0, 200 mM NaCl, and 1 mM TCEP). Pure fractions were pooled, concentrated and flash-frozen in liquid nitrogen and stored until further use.

Twa1 was also expressed as a His_6-SUMO fusion protein and purified using the same strategy with an initial Protino® Ni-IDA column affinity column (IMAC) followed by SEC with a HighLoad™ 26/600 Superdex™ 200 pg column. Only the buffer composition varied: LEW II buffer (20 mM tris pH 8.0, 500 mM NaCl, and 1 mM TCEP) and SEC II buffer (20 mM tris pH 8.0, 200 mM NaCl, and 1 mM TCEP) were used, and, instead of cleaving the His_6-SUMO tag on the column, tagged Twa1 was eluted using 4 CV of elution buffer (SEC II buffer + 500 mM imidazole) and tag cleavage was performed while dialyzing overnight against SEC II buffer.

For purification of co-expressed His_6-SUMO tagged RanBP9 together with Twa1 and His_6-SUMO tagged muskelin and its variants an optional AIEX step was utilized. After IMAC elution from a Protino® Ni-IDA column and in elution buffer (LEW II + 500 mM imidazole), tag cleavage was performed while dialyzing against MonoQ low salt I buffer (20 mM tris pH 8.0, 50 mM NaCl, and 1 mM TCEP). The dialysate was centrifuged for at least 20 min at 30,000 x g and applied to a MonoQ™ 10/100 GL column (Cytiva) in MonoQ low salt I buffer. The protein was eluted from the column using a 0-100% gradient with MonoQ high salt I buffer (20 mM tris 8.0, 1 M NaCl, and 1 mM TCEP) over 25 CV. Suitable fractions were pooled, concentrated, and used for size exclusion chromatography in SEC III buffer (20 mM Pipes pH 7.5, 200 mM NaCl, and 1 mM TCEP). Shorter muskelin constructs were purified as described earlier (1).

IMAC elution of His_6-Wdr26 from a 5 ml HisTrap crude column (Cytivia) with LEW III (30 mM HEPES 7.5, 500 mM NaCl, 10% glycerol, and 1 mM TCEP) was performed in elution buffer (LEW III + 500 mM imidazole). His_6-tag cleavage using TEV protease was performed while dialyzing in MonoQ low salt II buffer (30 mM HEPES pH 7.5, 100 mM NaCl, and 1 mM TCEP). Cleaved Wdr26 was subjected to AIEX with a MonoQ™ 10/100 column and eluted with a gradient with MonoQ high salt II buffer (30 mM HEPES pH7.5, 1 M NaCl, and 1 mM TCEP). As a final purification step a Superose™ 6 16/70 column (Cytivia) using SEC IV buffer (30 mM HEPES pH 7.5, 500 mM NaCl, and 1 mM TCEP) was employed.

For purification of CTLH complexes expressed in insect cells (T_{cat}, RT_{cat}, $RT\alpha_{cat}$, and $WRT\alpha_{cat}$), a two-step purification was used. An IMAC step using Ni-NTA beads (Macherey-Nagel) in a gravity column in LEW IV buffer (50 mM tris pH 9.0, 250 mM NaCl, 10% glycerol, 25 mM imidazole, and 1 mM TCEP) and elution buffer (50 mM tris pH 9.0, 500 mM NaCl, 10% glycerol, 250 mM imidazole, and 1 mM TCEP) was followed by a SEC step with a Superose™ 6 16/70 column in SEC V buffer (25 mM tris pH 9.0, 250 mM NaCl, 10% glycerol, and 1 mM TCEP).

Analytical size exclusion chromatography (aSEC) for interaction studies

Protein complexes were incubated on ice at a 1:1 molar ratio at suitable concentrations for at least 30 min. After centrifugation at 30,000 x g for at least 20 min, 500 µl of sample were applied in a suitable SEC buffer (same composition used earlier for purification) to a pre-equilibrated Superose™ 6 increase 10/300 GL column (Cytiva). Selected elution fractions were analyzed by SDS-PAGE.

Sodium dodecyl sulfate–polyacrylamide gel electrophoresis (SDS-PAGE)
Samples were diluted in 5x SDS sample buffer (250 mM tris pH 6.8, 50% glycerol, 500 mM DTT, 10% (w/v) SDS, 0.5% bromphenol blue), denatured at 95 °C for 5 min, and loaded either on 4-20% gradient Mini-PROTEAN® TGX™ precast gels (BioRad) or 15% hand cast gels which were inserted into a Mini-PROTEAN® Tetra cell chamber (BioRad) filled with running buffer (190 mM glycine, 25 mM tris, 0.1% SDS). For hand casting, running gel (15% acrylamide/bis-acrylamide solution, 375 mM tris pH 8.8, 0.1% SDS, freshly added: 0.1% APS and 0,4‰ TEMED) and stacking gel (5% acrylamide/bis-acrylamide solution, 125 mM tris pH 6.8, 0.1% SDS, freshly added: 0.1% APS and 1‰ TEMED) solutions were subsequently poured and polymerized between the glass plates of the Mini-PROTEAN® 3 Multi-Casting Chamber (BioRad). Separation of samples and of a molecular weight marker, PageRuler™ Prestained Protein Ladder (Thermo Scientific), were performed at 200 V for 50-60 min at room temperature. For staining, the gel was washed three times with boiling water, incubated for 5 min in hot G-250 staining solution (0.08‰ Coomassie Brilliant Blue G-250, 36 mM HCl) and destained using distilled water.

Native agarose gel electrophoresis (NAGE)
Purified muskelin constructs and RanBP9-Twa1 complex were thawed, centrifuged at 25,000 x g for 30 min, and the concentration was determined using the UV absorption at 280 nm. Proteins were diluted to a final concentration of 50 µM and incubated with their prospective binding partner for at least 30 min at room temperature. Samples were separated on freshly prepared 0.8% HEEO agarose gels for 2 h at 120 V at 4 °C in NAGE buffer (25 mM tris and 200 mM glycine). After separation, gels were incubated in staining solution (0.05% Coomassie R250, 50% methanol and 10% acetic acid) for at least 20 min, followed by destaining.

Blue native polyacrylamide gel electrophoresis (BN-PAGE)
BN-PAGE was performed according to the manufacturer's instructions (Life technologies™). CTLH subunits and complex assemblies were incubated for at least 1 h on ice at concentrations of 20 µM. Samples were diluted in 4x NativePAGE™ sample buffer (50 mM bis-tris pH 7.2, 50 mM NaCl, 10% (w/v) glycerol, 0.001% Ponceau S) and loaded on a NativePAGE™ Novex® 3–12% Bis-Tris gel (Invitrogen) which was placed in an XCell™ SureLock™ Mini-Cell. Additionally, the NativeMark™ Unstained Protein Standard (Invitrogen) was loaded as a molecular weight control. The cell was filled with anode buffer (50 mM bis-tris and 50 mM tricine, pH 6.8) and cathode buffer (anode buffer + 0.02% (w/v) Coomassie G-250) and proteins were separated in the cold room at 150 V for 60 min and 250 V for 30 min. For staining, the gel was incubated in fixation solution (40% methanol and 10% acetic acid) for at least 30 min and destained with destaining solution (10% ethanol and 5% acetic acid).

Crystallization, data collection and structure solution of Armc8β
SeMet-Armc8β crystals were obtained at 20 °C in a hanging drop vapor diffusion setup where 2 µl of protein at a concentration of 3 mg/ml were mixed with 6 µl of reservoir solution (100 mM MES pH 5.5, 150 mM $(NH_4)_2SO_4$ and 8% PEG 3350). For cryo-protection crystals were transferred to a cryo-solution (reservoir solution + 30% glycerol) and flash-cooled in liquid nitrogen. Diffraction data were collected at the European Synchrotron Radiation Facility (ESRF, Grenoble, France) at beamline ID23-1 (7) at the selenium edge (0.9737/0.9785 Å) on a PILATUS 6M-F detector (Dectris) at a temperature of 100 K using MXCuBE (8).
After integration and combination of four data sets collected from the same crystals with XDS (9) and Aimless (10) in the CCP4 suite interface (11, 12), the structure of Armc8β was solved using the datasets collected at 0.9785 A by the single wavelength anomalous dispersion method (SAD) utilizing the automated experimental phasing pipeline CRANK2 (13) in CCP4. For model building in Coot (14), only the best data set collected at 0.9737 Å was used, which was anisotropy corrected with the STARANISO server (15). Refinement was carried out with autoBuster (16) and Phenix (17, 18).

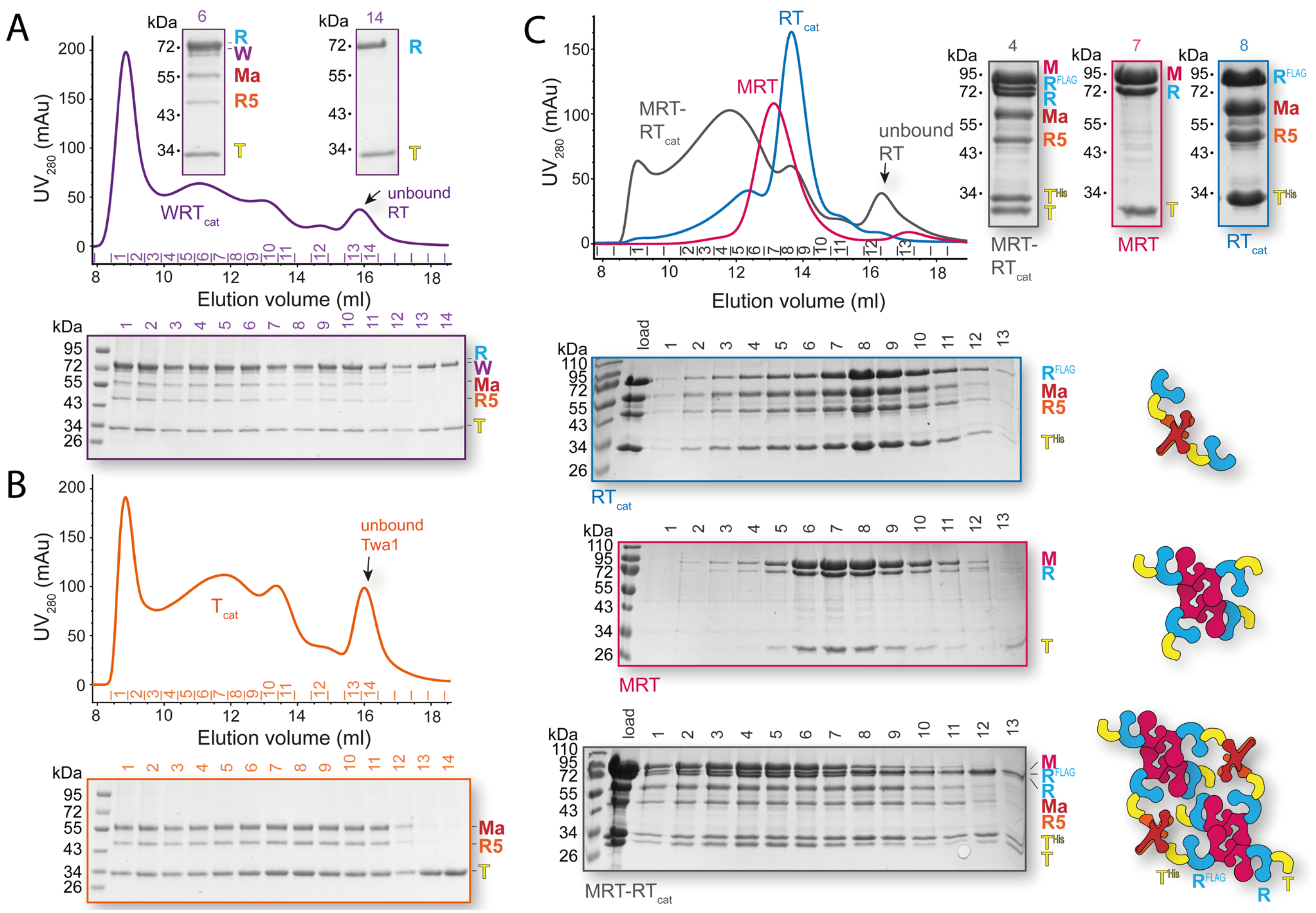

Figure S1: SDS-gel analyses of fractions from selected chromatograms.

The indicated fractions were analyzed to assess the separation of purified and assembled CTLH complexes by aSEC and to substantiate the assignment of the indicated elutions (arrows) of Twa1 and the R9T-complex.

(A) Analysis of the same purification as used for the WRT_{cat} complex in Figure 2A.

(B) Representative analysis for the T_{cat} complex as used in the assembly studies in Figure S3.

(C) Analysis of the three chromatograms from Figure 3G and models illustrating possible complex compositions of the respective main peak.

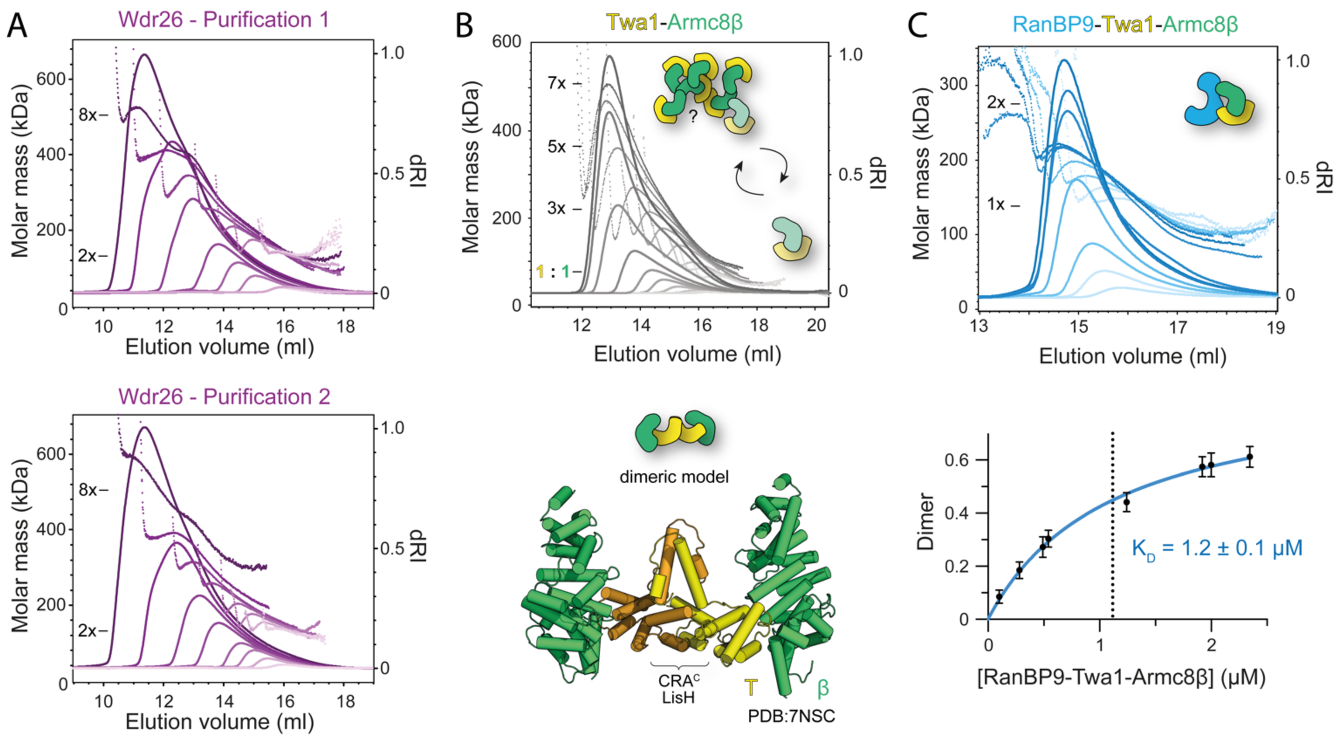

Figure S2: Dynamic oligomerization of Wdr26 as well as the Tβ and RTβ complexes.

Molar mass profiles of the SEC-MALS analysis are plotted over the entire elution range.

(A) Analysis of Wdr26 oligomerization as in Figure 2B from two dilution series derived from different purification batches.

(B) Analysis of the Tβ complex as shown in Figure 6A. Model of the Twa1 dimer based on Figure 2D. Binding of Armc8β based on an overlay with PDB entry 7NSC cannot explain the disruption of Twa1 dimerization as must have happened in the complex with a 1:1 binding stoichiometry.

(C) Analysis of the ternary RTβ-complex as shown in Figure 6C and analysis of its self-association (similar to those described in Figures 2B, 4A and 5A).

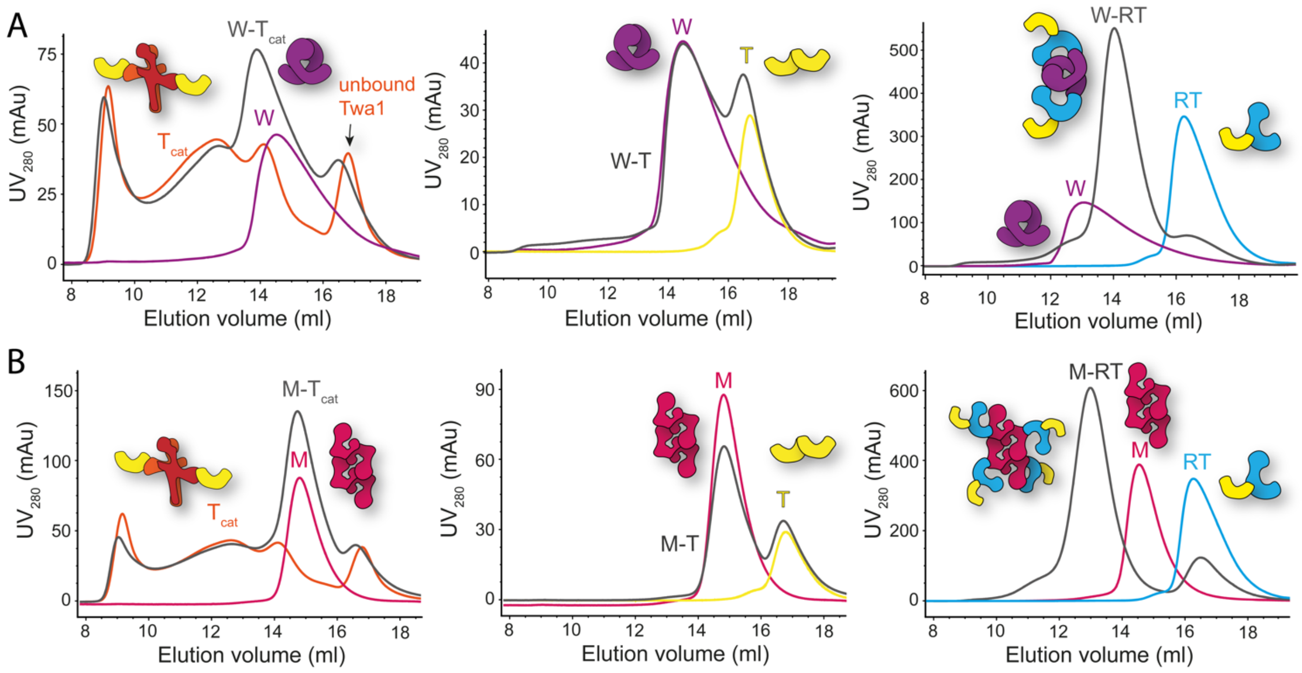

Figure S3: Wdr26 and muskelin both bind to RanBP9.

UV280 elution profiles of aSEC runs performed on a Superose^{TM} 6 increase 10/300 GL column as described in Figure S1. **(A)** Wdr26 and **(B)** muskelin both bind to RanBP9 based on complex assembly studies (gray) involving the ternary T_{cat} complex (orange, compare Figure S1B), Twa1 (yellow) and the binary RT-complex (blue).

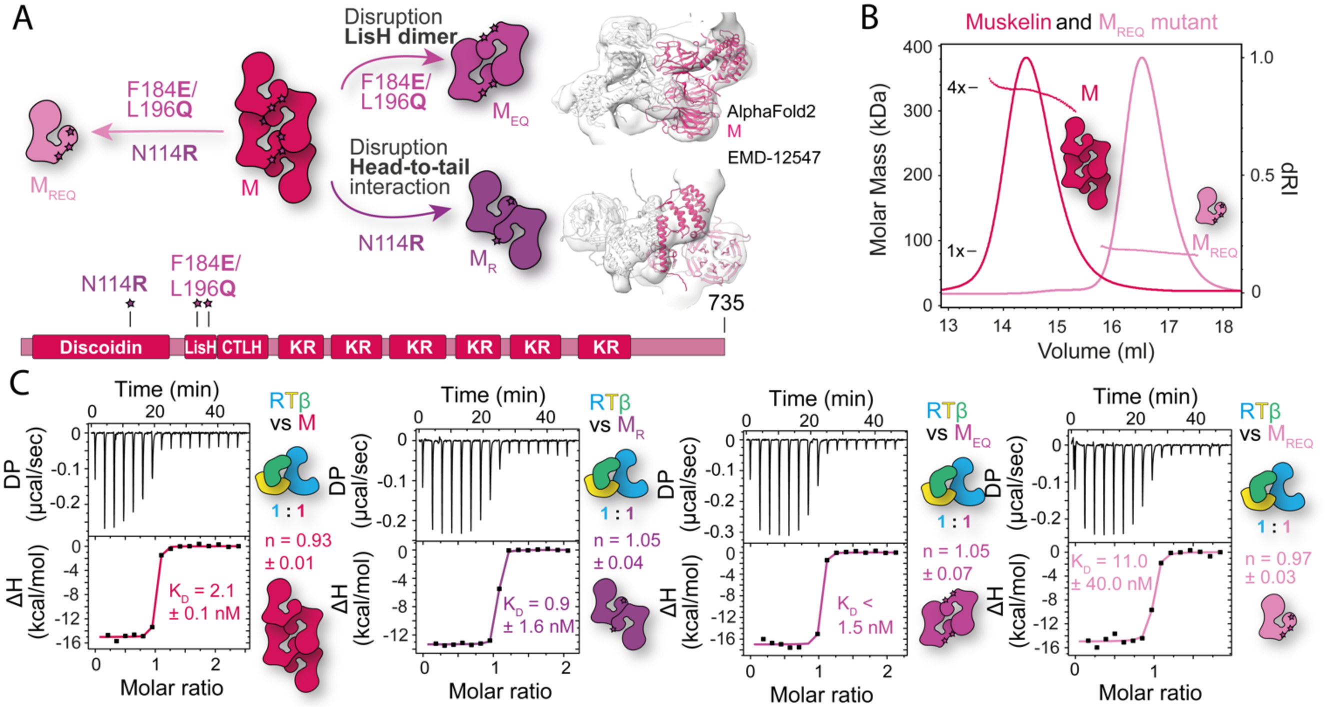

Figure S4: Muskelin oligomerization and binding to RTβ.

(A) LisH-mediated dimerization is disrupted by the F184E and L196Q mutations in the LisH motif, while the N114R mutation in the discoidin domain abolishes the head to tail interaction (1). Models of the resulting dimers were generated by fitting the AlphaFold2 structure prediction (19) into the muskelin cryo-EM map of EMD-12547 (see also Figure 3F).

(B) Oligomeric state of muskelin. SEC-MALS analysis of muskelin and the M_{REQ} variant (N114R, F184E, L196Q) demonstrate disruption of muskelin's tetrameric assembly (measured M_W: 329 ± 8 kDa, theoretical M_W: 339 kDa) in the triple mutant (measured MW: 84.4 ± 2.0 kDa, theoretical MW: 84.7 kDa).

(C) ITC studies of titrating the ternary RTβ complex to muskelin and its oligomerization-impaired mutants (M_R, M_{EQ}, M_{REQ}). Similar binding ratios were obtained in ITC experiments of the binary RT complex with muskelin (Figure 4C) and its mutants (data not shown).

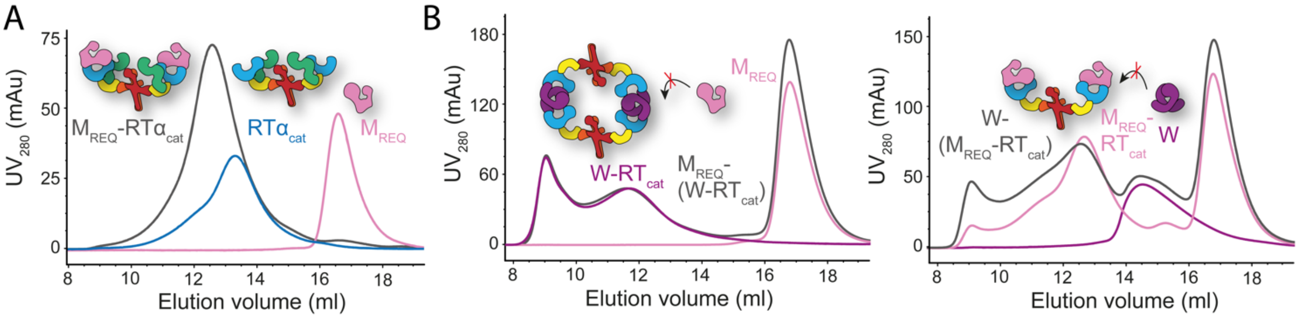

Figure S5: Mutually exclusive binding of Wdr26 and muskelin to the CTLH complex.

To circumvent complications of complex oligomerization because of the tetrameric muskelin architecture, its monomeric mutant M_{REQ} (see Figure S4A) was used for assembly studies.

(A) Assembly of M_{REQ} with the quinary co-expressed $RT\alpha_{cat}$ complex. Control experiment for Figure 3A.

(B) The quaternary RT_{cat} complex was pre-assembled with Wdr26 (left) or M_{REQ} (right), and M_{REQ} (left) or Wdr26 (right) was added.

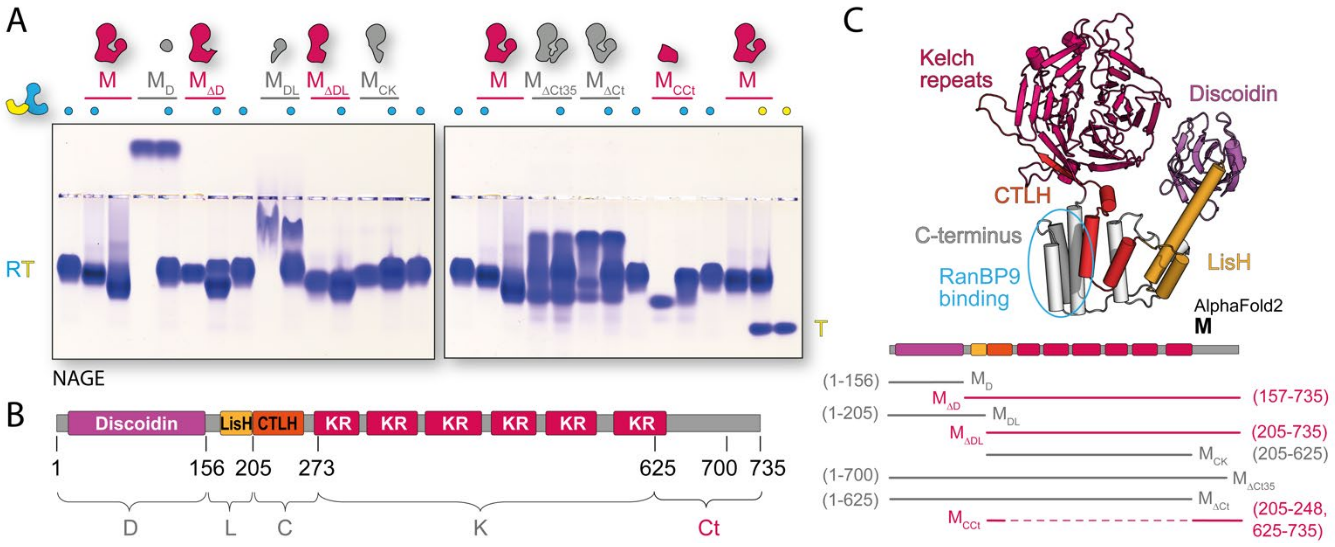

Figure S6: Determination of the RanBP9 binding site in muskelin.

(A) Binding of different muskelin deletion constructs were tested for interaction with the RT complex by native agarose gel electrophoresis (NAGE), as described in Figure 3D.

(B) Domain architecture of muskelin. Boundaries of constructs used for NAGE are shown.

(C) AlphaFold2 predicted structure of muskelin (19) is colored based on its domain architecture in (B). The constructs as analyzed in (A) are shown below the domain architecture. RT-interacting constructs are highlighted in magenta.

Table S1. Data collection and refinement statistics of Armc8β.

**Data collection**		
	**SeMet-Armc8β**	**SAD SeMet-Armc8β**
PDB ID	8A1I	-
Wavelength (Å)	0.9737	0.9785
Space group	$P\ 1\ 2_1\ 1$	$P\ 1\ 2_1\ 1$
Cell dimensions		
a, *b*, *c* (Å)	65.64, 148.18, 67.14	65.64, 148.19, 67.15
α, β, γ (°)	90.00, 90.57, 90.00	90.00, 90.57, 90.00
Resolution limits (Å)	47.17-2.69 (2.92-2.69)	49.76-3.00 (3.18-3.00)
Observed reflections	128,903 (7,817)	462,605 (29,261)
Unique reflections	26,256 (1,313)	25,703 (4,137)
$^{a}R_{merge}$	0.166 (1.658)	0.355 (1.905)
$^{b}R_{pim}$	0.085 (0.739)	0.082 (0.771)
$CC_{1/2}$	0.993 (0.363)	0.997 (0.427)
$^{c}\langle I/\sigma I\rangle$	7.9 (1.1)	9.8 (1.1)
Overall completeness spherical/elliptical (highest resolution shell)	73.9/90.7 (17.0/47.9)	100.0 (100.0)
Multiplicity	4.9 (6.0)	18.0 (7.1)
Wilson B-factor (Å^2)	75.7	53.3
**Phasing**		
Method		SAD
Anomalous completeness		99.9 (99.7)
Anomalous multiplicity		9.0 (3.6)
FOM (phasing)		0.2091
FOM (density modification+model building)		0.7901
**Refinement**		
No. reflections	26,174	
$^{d}R_{\mathrm{work}}$ / $^{e}R_{\mathrm{free}}$	0.2137/0.2484	
No. of atoms	8,684	
Protein	8,584	
Ligands	62	
Solvent	38	
Protein residues	1,096	
Rmachandran statistics: favored/allowed (%)	99.17/0.83	
Clashscore	4.33	
Overall B-factor (Å^2)	85.4	
RMS deviations in		
Bonds (Å)	0.004	
Angles (°)	0.70	

[a]$R_{sym} = \Sigma_{hkl}\Sigma_i \mid I_i - \langle I \rangle \mid / \Sigma_{hkl}\Sigma_i I_i$ where I_i is the ith measurement and $\langle I \rangle$ is the weighted mean of all measurements of *I*.

[b]$R_{pim} = \Sigma_{hkl} 1/(N-1)^{½} \Sigma_i \mid I_i(hkl) - \overline{I(hkl)} \mid / \Sigma_{hkl}\Sigma_i I(hkl)$, where N is the redundancy of the data and $I(hkl)$ the average intensity.

[c]$\langle I / \sigma I \rangle$ indicates the average of the intensity divided by its standard deviation.

[d]$R_{work} = \Sigma_{hkl} ||F_o| - |F_c|| / \Sigma_{hkl}|F_o|$ where F_o and F_c are the observed and calculated structure factor amplitudes.

[e]R_{free} same as R for 5% of the data randomly omitted from the refinement. The number of reflections includes the R_{free} subset.

[f]Ramachandran statistics were calculated with MolProbity in PHENIX.

Numbers in parentheses refer to the respective highest resolution data shell in the dataset.

Movie S1 (separate file): Morph of the three different conformations observed in the crystal structure of Armc8β. Chain p, i and a of PDB: 8A1I were morphed and visualized using the PyMOL Molecular Graphics System (Schrödinger, LLC) software.

SI References

1. Delto, C. F., Heisler, F. F., Kuper, J., Sander, B., Kneussel, M., and Schindelin, H. (2015) The LisH motif of muskelin is crucial for oligomerization and governs intracellular localization. *Struct. Lond. Engl. 1993*. **23**, 364–373
2. Pfirrmann, T., Lokapally, A., Andréasson, C., Ljungdahl, P., and Hollemann, T. (2013) SOMA: A Single Oligonucleotide Mutagenesis and Cloning Approach. *PLOS ONE*. **8**, e64870
3. Li, M. Z., and Elledge, S. J. (2007) Harnessing homologous recombination in vitro to generate recombinant DNA via SLIC. *Nat. Methods*. **4**, 251–256
4. Berger, I., Fitzgerald, D. J., and Richmond, T. J. (2004) Baculovirus expression system for heterologous multiprotein complexes. *Nat. Biotechnol.* **22**, 1583–1587
5. Bieniossek, C., Imasaki, T., Takagi, Y., and Berger, I. (2012) MultiBac: expanding the research toolbox for multiprotein complexes. *Trends Biochem. Sci.* **37**, 49–57
6. Bleckmann, M., Schürig, M., Chen, F.-F., Yen, Z.-Z., Lindemann, N., Meyer, S., Spehr, J., and Heuvel, J. van den (2016) Identification of Essential Genetic Baculoviral Elements for Recombinant Protein Expression by Transactivation in Sf21 Insect Cells. *PLOS ONE*. **11**, e0149424
7. Nurizzo, D., Mairs, T., Guijarro, M., Rey, V., Meyer, J., Fajardo, P., Chavanne, J., Biasci, J. C., McSweeney, S., and Mitchell, E. (2006) The ID23-1 structural biology beamline at the ESRF. *J. Synchrotron Radiat.* **13**, 227–238
8. Gabadinho, J., Beteva, A., Guijarro, M., Rey-Bakaikoa, V., Spruce, D., Bowler, M. W., Brockhauser, S., Flot, D., Gordon, E. J., Hall, D. R., Lavault, B., McCarthy, A. A., McCarthy, J., Mitchell, E., Monaco, S., Mueller-Dieckmann, C., Nurizzo, D., Ravelli, R. B. G., Thibault, X., Walsh, M. A., Leonard, G. A., and McSweeney, S. M. (2010) MxCuBE: a synchrotron beamline control environment customized for macromolecular crystallography experiments. *J. Synchrotron Radiat.* **17**, 700–707
9. Kabsch, W. (2010) XDS. *Acta Crystallogr. D Biol. Crystallogr.* **66**, 125–132
10. Evans, P. R., and Murshudov, G. N. (2013) How good are my data and what is the resolution? *Acta Crystallogr. D Biol. Crystallogr.* **69**, 1204–1214
11. Potterton, E., Briggs, P., Turkenburg, M., and Dodson, E. (2003) A graphical user interface to the CCP4 program suite. *Acta Crystallogr. D Biol. Crystallogr.* **59**, 1131–1137
12. Winn, M. D., Ballard, C. C., Cowtan, K. D., Dodson, E. J., Emsley, P., Evans, P. R., Keegan, R. M., Krissinel, E. B., Leslie, A. G. W., McCoy, A., McNicholas, S. J., Murshudov, G. N., Pannu, N. S., Potterton, E. A., Powell, H. R., Read, R. J., Vagin, A., and Wilson, K. S. (2011) Overview of the CCP4 suite and current developments. *Acta Crystallogr. D Biol. Crystallogr.* **67**, 235–242
13. Skubák, P., and Pannu, N. S. (2013) Automatic protein structure solution from weak X-ray data. *Nat. Commun.* **4**, 2777
14. Emsley, P., Lohkamp, B., Scott, W. G., and Cowtan, K. (2010) Features and development of Coot. *Acta Crystallogr. D Biol. Crystallogr.* **66**, 486–501
15. Tickle, I. J., Flensburg, C., Keller, P., and Paciorek, W., Sharff, A., Vonrhein, C., Bricogne, G. (2018) *STARANISO*, Global Phasing Ltd., Cambridge, United Kingdom
16. Bricogne, G., Blanc, E., Brandl, M., Flensburg, C., Keller, P., Paciorek, W., Roversi, P., Sharff, A., Smart, O. S., Vonrhein, C., and Womack, T. O. (2017) *BUSTER version 2.10.4.*, Global Phasing Ltd., Cambridge, United Kingdom
17. Afonine, P. V., Grosse-Kunstleve, R. W., Echols, N., Headd, J. J., Moriarty, N. W., Mustyakimov, M., Terwilliger, T. C., Urzhumtsev, A., Zwart, P. H., and Adams, P. D. (2012) Towards automated crystallographic structure refinement with phenix.refine. *Acta Crystallogr. D Biol. Crystallogr.* **68**, 352–367
18. Liebschner, D., Afonine, P. V., Baker, M. L., Bunkóczi, G., Chen, V. B., Croll, T. I., Hintze, B., Hung, L. W., Jain, S., McCoy, A. J., Moriarty, N. W., Oeffner, R. D., Poon, B. K., Prisant, M. G., Read, R. J., Richardson, J. S., Richardson, D. C., Sammito, M. D., Sobolev, O. V., Stockwell, D. H., Terwilliger, T. C., Urzhumtsev, A. G., Videau, L. L., Williams, C. J., and Adams, P. D. (2019) Macromolecular structure determination using X-rays, neutrons and electrons: recent developments in Phenix. *Acta Crystallogr. Sect. Struct. Biol.* **75**, 861–877
19. Varadi, M., Anyango, S., Deshpande, M., Nair, S., Natassia, C., Yordanova, G., Yuan, D., Stroe, O., Wood, G., Laydon, A., Žídek, A., Green, T., Tunyasuvunakool, K., Petersen, S., Jumper, J., Clancy, E., Green, R., Vora, A., Lutfi, M., Figurnov, M., Cowie, A., Hobbs, N., Kohli, P., Kleywegt, G., Birney, E., Hassabis, D., and Velankar, S. (2022) AlphaFold Protein Structure Database: massively expanding the structural coverage of protein-sequence space with high-accuracy models. *Nucleic Acids Res.* **50**, D439–D444
